# Octopus-inspired muscle activation unlocks high-DOF motion from a single actuator

**DOI:** 10.1126/sciadv.aei4282

**Published:** 2026-09-23

**Authors:** Wenci Xin, Zhiqiang Tang, Peiyi Wang, Lei He, Linxin Hou, Lin Zhao, Zhexin Xie, Cecilia Laschi

**Affiliations:** ^1^ Department of Mechanical Engineering, National University of Singapore, 117575, Singapore.; ^2^ Advanced Robotics Centre, National University of Singapore, 117575, Singapore.; ^3^ School of Mechanical Engineering, Southeast University, Nanjing, 211189, China.; ^4^ Department of Electrical and Computer Engineering, National University of Singapore, 117575, Singapore.; ^5^ Shenzhen Key Laboratory of Biomimetic Robotics and Intelligent Systems, Department of Mechanical and Energy Engineering, Southern University of Science and Technology, 518055, Shenzhen, China.; ^6^ Institute for Robotics, Southern University of Science and Technology, Shenzhen, 518055, China.

## Abstract

While soft robots leverage compliant materials to offer infinite degrees of freedom (DOFs), achieving dexterous motions usually requires numerous actuators, making the robot bulky, complex to assemble, and discontinuously segmented. Here, inspired by the octopus neuromuscular junctions (NMJs), we present similar wire bonding junctions (WBJs) that enable partitioned activation on a single shape memory alloy (SMA) actuator. By utilizing WBJs, soft robots achieve continuous deformation with approximately twice the workspace and manipulability of traditional stacked designs. We demonstrated that modulating voltage across these junctions allows the SMA to actuate variable lengths, maintaining high dexterity while reducing energy consumption up to 40%. With WBJs, one single SMA actuator could master both soft arm manipulator and its end effector. This compact, lightweight architecture enables the soft robot arm to be rolled for low-encumbrance drone deployment and confined-space sampling. Our results demonstrate a new actuation paradigm for achieving high DOF, offering a compact, efficient blueprint for soft robot design.

## INTRODUCTION

Octopus arms exhibit remarkable versatility across diverse conditions, from retrieving objects in confined spaces to rapidly capturing preys in complex underwater environments (*1*–*4*). Such versatility arises from the arm’s boneless, continuously deformable body, in which spatially distributed muscle activation enables localized control of shape and mechanics, producing behaviors unattainable in vertebrates (*5*, *6*). The inability of articulated robotic systems to replicate these capabilities has motivated the development of soft robots that overcome fundamental limitations (*7*, *8*), their continuously deformable bodies, in principle, provide virtually unlimited DOF and broad opportunities for adaptive interaction with the environment (*9*–*15*)

Despite the high DOF afforded by soft robots, actuation remains a key bottleneck in fully exploiting this capability (*16*–*18*). To increase DOF and dexterity, most existing soft robots rely on increasing the number of serial actuators like pneumatic chambers and cable-driven units (*19*–*23*), resulting in the fixtures at both ends of each actuator interrupt mechanical continuity along the structure, an issue that becomes increasingly severe as robot size decreases (*24*). Increasing the number of controllable DOF additionally requires further actuators, routing structures and electrical interconnections. These approaches also introduce fabrication complexity (*25*, *26*), structural discontinuity, performance inconsistency, and impose growing demands on power and regulation (*27*, *28*). As a result, many soft robotic systems still depend heavily on passive deformation and fall short of the dexterity observed in animals.

**Fig. 1.**
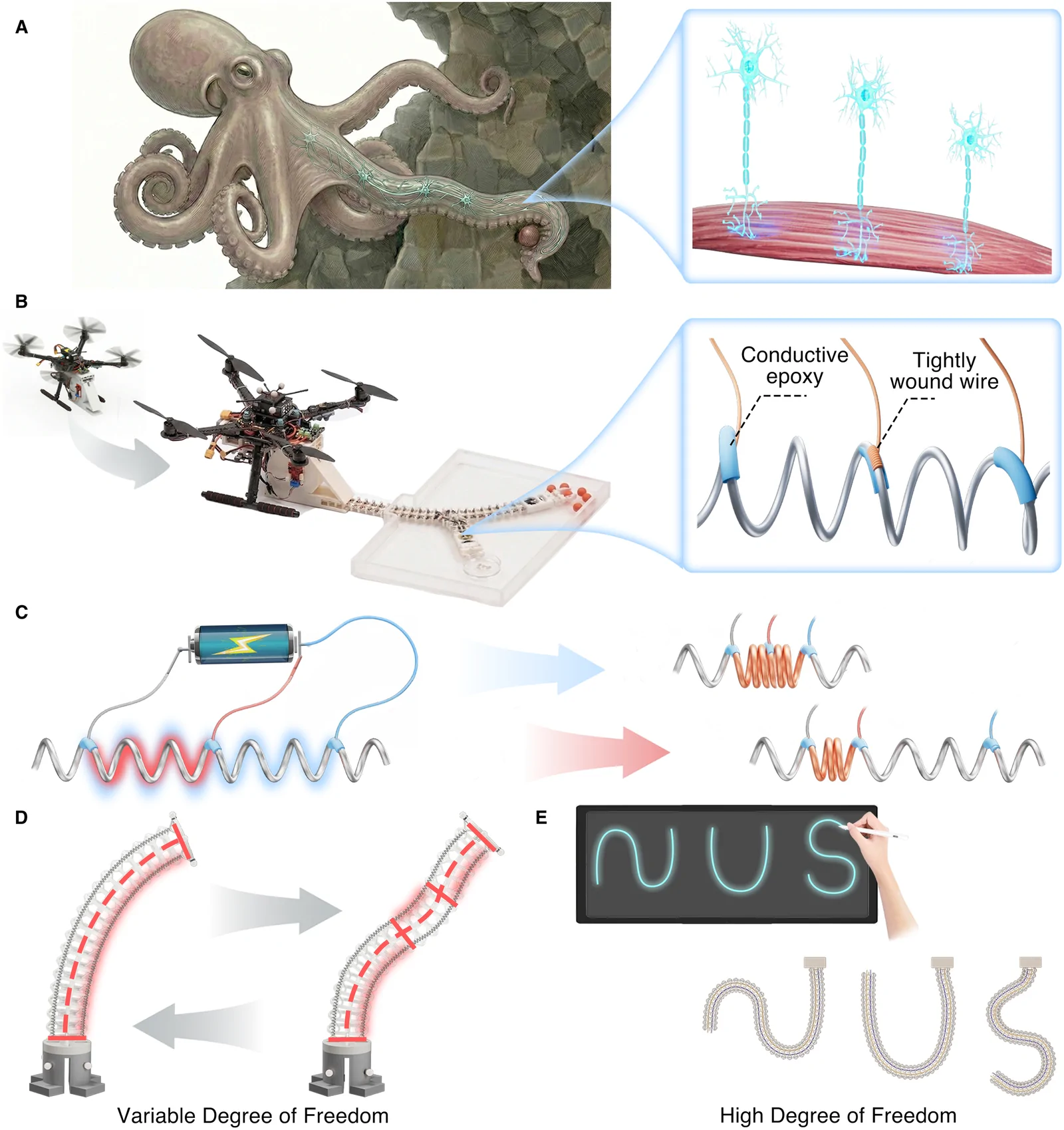
Bioinspiration of WBJ actuation and capabilities achieved by the local actuation. (**A**) The serial arrangement of neuromuscular junctions (NMJs) enables the octopus arm to perform complex manipulations in confined spaces using functionally individual muscle units along its length (**B**) The serial arrangement of wire bonding junctions (WBJs) enables the soft robotic arm to complete multiple tasks in confined spaces using two shape memory alloy (SMA) actuators along the arm. (**C**) Actuation of the wire bonding junction enables the SMA get partially actuate. Based on the proposed actuation method, the soft arm is capable of (**D**) varying degree of freedoms and (**E**) dexterous shape changing with high degree of freedoms.

This limitation reflects a deeper inheritance from vertebrate-inspired actuation. In vertebrates, slender skeletal muscles are activated to contract simultaneously by the neural signals through neuromuscular junctions (NMJs) to generate large forces for motion of discrete joints (*29*–*31*). Robotic actuation has largely followed the same logic: one actuator, one dominant mechanical output, and one local degree of freedom. Octopus arms, by contrast, follow a different neuromuscular architecture. Octopus arm muscles lack clear segmentation and instead form continuous muscle bundles densely innervated by numerous NMJs, their muscle cells are small, electrically compact, and allowing dense innervation, enabling highly localized contraction without long-range propagation along individual muscle fibers (*32*–*35*). Signals delivered through NMJs activate local regions rather than entire fibers, allowing localized control of contraction (Fig. 1A) with high spatial resolution along continuous muscle bundles (*36*, *37*). This organization suggests an alternative actuation paradigm: high-DOF behavior can emerge not from multiplying discrete actuators, but from spatially programming actuation within a continuous one.

Here, inspired by the octopus neuromuscular system, we present an actuation strategy that enables high–DOF motion from a single actuator. We employ shape memory alloy (SMA) as a model actuator owing to its intrinsic electrical conductivity, which facilitates implementation of the proposed strategy. By incorporating wire-bonding junctions (WBJs) that functionally emulate neuromuscular junctions (NMJs) along a single spring-shaped SMA actuator (Fig. 1B), and applying electrical potentials selectively across these junctions, actuation can be localized to different regions of the same SMA actuator, allowing spatially programmable contraction within a continuous soft body (Fig. 1C). In addition, when applying the SMA actuator as an artificial muscle to drive robotic arm motion, actively floating selected WBJs enables dynamic reconfiguration of effective segmentation and controllable DOF of the soft arm, achieving behaviors analogous to those used by animals to reduce control complexity and energy consumption (Fig. 1D). This strategy establishes a scalable route toward mechanically continuous, high-DOF soft robots without relying on large numbers of discrete actuators or complex transmission architectures, while enabling rich shape transformations and task versatility from a compact actuation system (Fig. 1E).

## RESULTS

### Structure and performance of Wire Bonding Junctions (WBJs)

In the SMA actuator, WBJs are introduced to assign distinct electrical potentials to different points of the actuator, requiring each junction to remain both mechanically and electrically stable during actuation. Figure 1B illustrates the internal structure of WBJ. To achieve sufficient stroke for large deformation, the SMA actuators are pre-trained into spring like geometry.

**Fig. 2.**
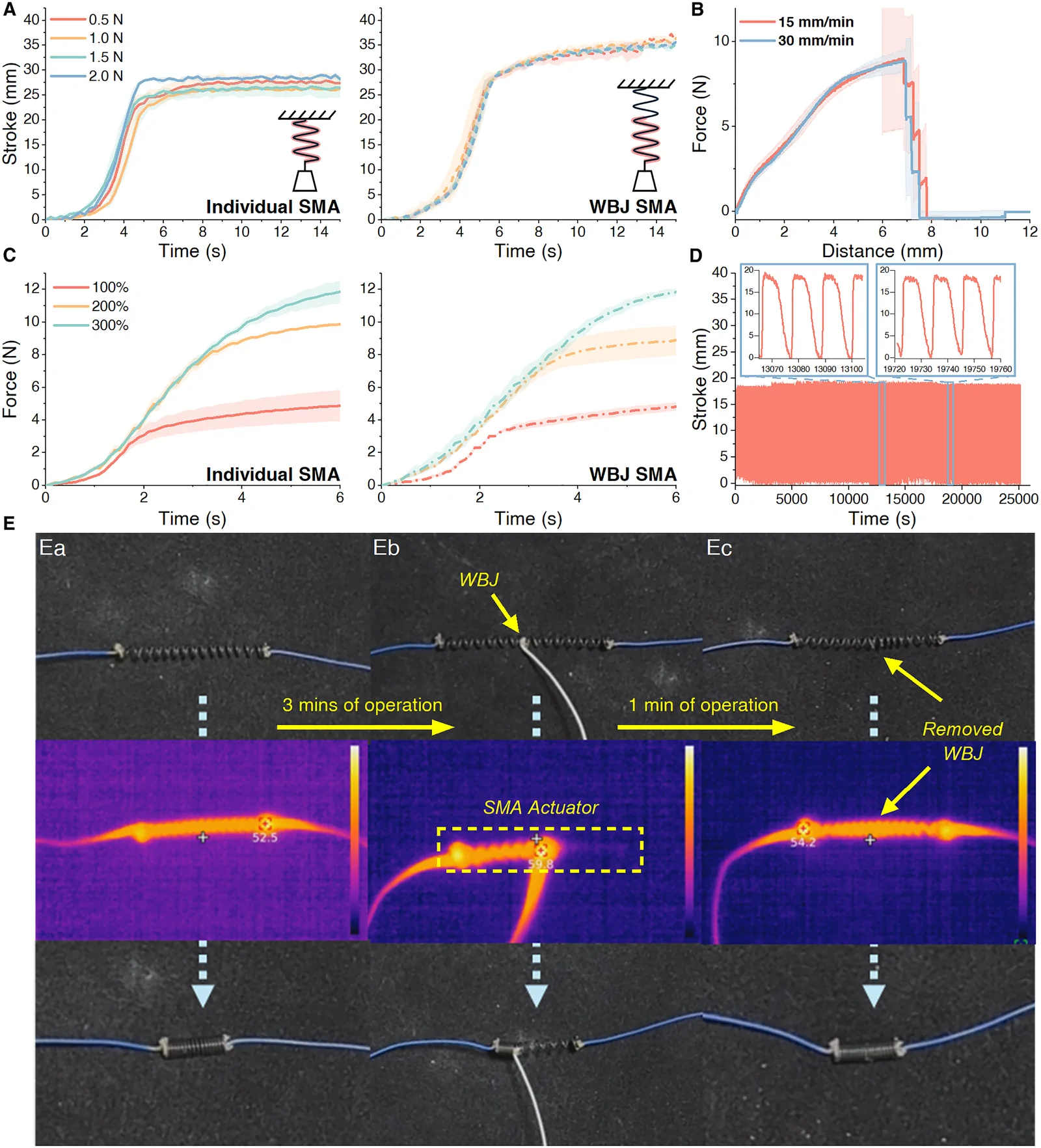
Characterization of the WBJ actuation method. (**A**) Stroke performance between the WBJ-segmented and individual SMA actuator under difference load (actuation current: 2A). (**B**) Mechanical strength of the WBJ under different loading test. (**C**) Payload performance between the WBJ-segmented and individual SMA actuator under difference elongating rate (actuation current: 2A). (**D**) Durability test of the WBJ segmented actuator. (**E**) Performance of the SMA actuator after the addition and removal of the WBJ, a process requiring only a few minutes.

To compare the performance of WBJ-segmented and individual SMA actuators, we characterized the actuator behavior under two conditions: contraction under a fixed payload and at a fixed length, measuring lifting stroke and contraction force, respectively (fig. S1). Under a constant current actuation of 2 A, the individual SMA actuator exhibits similar behavior across different payloads, lifting the load during the first 5.5 s before reaching a fully contracted state. By contrast, although the WBJ-segmented SMA actuator initially follows a similar contraction profile and lifts the payload to a comparable height, differing by 1.97 mm, it subsequently undergoes additional continuous contraction (Fig. 2A). This secondary contraction arises from heat conduction between the actively actuated SMA segment and adjacent inactive segments (fig. S2). As the current decreases, this heat conduction effect becomes more pronounced, leading to contraction beyond the capacity of the individually actuated SMA segment, with additional displacements of 5.06 mm and 12.3 mm observed at actuation currents of 1.5 A and 1.0 A, respectively (fig. S2A). Similarly, the force responses of the two actuators exhibit comparable trends. Because the actuator was fixed only at its two ends (fig. S1), heat conduction from the activated segment to adjacent inactive regions resulted in an average increase of 0.31 N in contraction force at a driving current of 1 A (fig. S2). At higher actuation levels, the remaining cases show reduced contraction force, which can be attributed to the spring-like resistance of the non-actuated SMA segments (Fig. 2C and fig. S2B). This artifact was absent in the subsequent robotic experiments, where the actuator was mechanically supported at the WBJ points rather than only at its two ends.

To assess reliability, we evaluated the mechanical strength and repeatability of the WBJs. Tensile tests were conducted at pulling speeds of 15 and 30 mm/min, in which the SMA actuator was fixed while the bonded wire was pulled to load the WBJ (fig. S3A). In both cases, failure occurred at forces of 9 N, with rupture observed at a displacement of 7 mm (Fig. 2B). Notably, the breaking point occurs at the electrical wires instead of WBJs (fig. S3, B and C). The repeatability of the WBJs was evaluated under cyclic actuation consisting of 1.5 s of activation followed by 10 s of active wind cooling. The WBJs maintained consistent performance over more than 2000 actuation cycles.

Owing to its simple structure, the fabrication of a WBJ is straightforward, requiring approximately 3 minutes for addition and 1 minute for removal, including only wire winding and epoxy encapsulation (movie S1). The relative independence of individual WBJs ensures that their addition or removal does not interfere with other junctions and can be performed at any stage of operation. To evaluate the impact of WBJ modification, we tested the thermal response of the same SMA actuator before and after adding or removing WBJs (Fig. 2E and movie S2). Even with two WBJs already present at the actuator ends, the addition of an extra WBJ does not affect the operation of the existing ones. Similarly, because WBJs are attached through a noninvasive bonding process, their removal does not degrade actuator performance. Even if there is residual conductive epoxy, the alloy-based actuator will automatically short-circuit it to ensure performance.

### Soft robot performance improvement compared with individual actuators

**Fig. 3.**
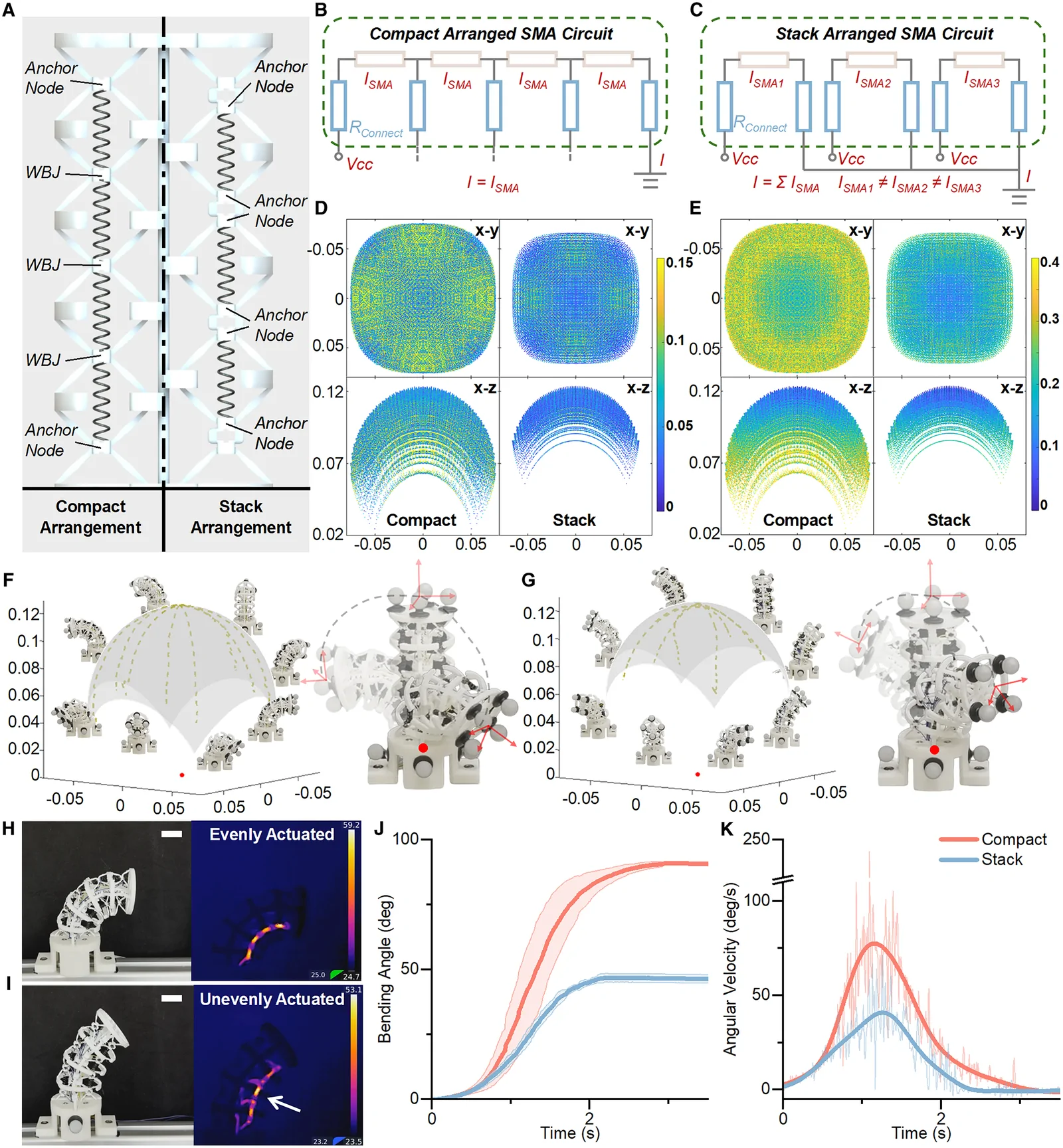
Comparison of the WBJ-enabled compact arrangement and individual SMAs for stack arrangement. (**A**) Schematics for the compact and stack actuator arrangement. (**B**) The working circuit of the series connected compact arrangement. (**C**) The working circuit of the parallel connected stack arrangement. (**D**) The manipulability of compact and stack arrangement in terms of linear velocity. (**E**) The manipulability of compact and stack arrangement in terms of angular velocity. (**F**) Experimental workspace test of the compact arrangement. (**G**) Experimental workspace test of the stack arrangement. (**H**) Bending movement of the compact arrangement and its thermal response. (**I**) Bending movement of the stack arrangement and its thermal response (scale bar: 2 cm). (**J**) Temporal profiles of the robot’s bending angle. (**K**) Temporal profiles of the robot’s angular velocity.

To evaluate the impact of WBJs at the system level, we further characterized performance improvements in soft robots embedding SMA actuators with WBJs. The experimental platform consists of a 3D-printed soft robotic arm with a fixed-length central backbone, surrounded by four SMA actuators arranged at 90° intervals. Two actuator configurations were compared: a compact arrangement, in which actuators are seamlessly connected via WBJs, and a stacked arrangement, which uses conventionally distributed individual SMA actuators (Fig. 3A). Mechanically, the stacked arrangement requires additional space for end anchoring and electrical isolation, resulting in only three active segments over a total length of 100 mm, whereas the compact arrangement accommodates four active segments within the same length. The intrinsic electrical continuity between active segments in the compact arrangement enables uniform electronic performance, such that the actuation current is identical across segments and the main circuit carries a single constant current (Fig. 3B). By contrast, the stacked arrangement operates as a parallel circuit and requires accumulated current among multiple branches from the power supply. Due to fabrication variability, particularly connect resistance which dominates given the low resistance of SMA actuators, the current delivered to each branch in the stacked configuration becomes uneven, leading to variation in actuation (Fig. 3C).

On the basis of kinematic model, we analyzed the differences in manipulability across the workspace for the two configurations in terms of both linear and angular velocities by introducing the ratio of the minor to major axes of the manipulation ellipsoid. The closer the value is to 1, the more maneuverable the robot is. Figure 3, D and E illustrate how the two actuator arrangements influence the linear and angular performance of the robotic arm, respectively. The compact arrangement achieves a substantially larger workspace, with a calculated volume of approximately 4.31 × 10^5^ mm^3^ by convex hull, compared with the shallower and smaller workspace of the stacked arrangement, which has a volume of 1.48 × 10^5^ mm^3^. Among the sampled configurations, the maximum manipulability ratios reach 0.271 for the compact arrangement and 0.158 for the stacked arrangement in terms of linear velocity, and 0.584 and 0.336, respectively, for angular velocity. After interpolating the sampled workspace of the compact arrangement onto that of the stacked arrangement, the compact configuration exhibits average improvements of 2.11 and 1.47 times in linear and angular manipulability, respectively.

We next conducted experiments to validate the workspace analysis. Robotic arms incorporating the two actuator configurations were fabricated and tested. Each of the four actuator series was actuated individually and in combination with its adjacent actuator, yielding eight distinct motion paths that delineate the outer boundary of the workspace (Fig. 3, F and G). Across all motion paths, the maximum displacements along the *x*, *y*, and *z* axes for the compact arrangement reached 70.0 mm, 72.0 mm, and 85.4 mm, respectively. In contrast, the stacked arrangement achieved smaller maximum displacements of 59.2 mm, 51.9 mm, and 57.7 mm along the corresponding axes. Because the actuation was performed in an open-loop manner, the diagonal paths were not linear but instead exhibited curvature due to differences in contraction speed between actuators, an effect that was particularly pronounced in the stacked arrangement. Comparing the measured paths with the theoretically derived workspace boundaries, the compact arrangement exhibited an average positional error of 4.4 mm, whereas the stack arrangement showed a larger error of 8.1 mm.

The thermal response of the two configurations is further evaluated during bending (Fig. 3, H and I). To eliminate current division, the stacked SMA actuators were connected in series. Under identical driving conditions, the WBJ-enabled compact arrangement achieved an angle of 91°, whereas the traditional stacked arrangement reached only 46°. Although the contraction performance of active segments was comparable, thermal imaging revealed distinct differences in heat distribution. The WBJ-enabled actuator maintained a nearly uniform temperature profile throughout actuation, whereas the stacked configuration exhibited localized heat accumulation, particularly in the second actuator (movie S3). These thermal differences were accompanied by distinct bending dynamics, as reflected in the asymmetric temporal profiles of the bending angle and angular velocity (Fig. 3, J and K). The nonuniform response became more pronounced when the stacked SMA actuators were connected in parallel, producing a bimodal angular velocity profile even under constant voltage actuation (fig. S4, A and S4B). A stacked actuator with the same total SMA length was also evaluated and exhibited both a lower bending speed and a smaller bending angle (fig. S4C).

### Actively changeable DOF

**Fig. 4.**
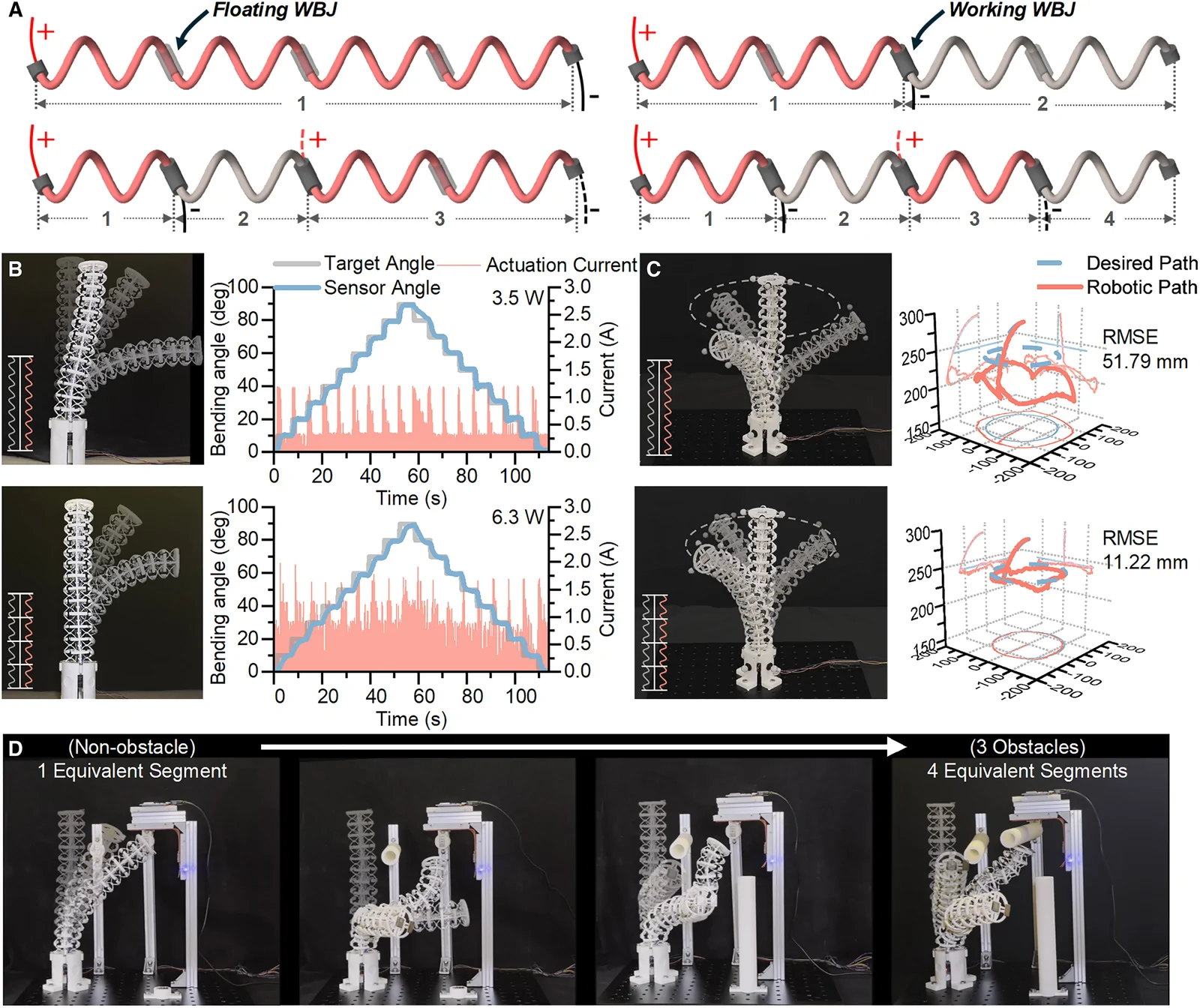
Variable DOF achieved by the WBJ actuation. (**A**) Schematic of the working principle for achieving variable DOF by the WBJ actuation. (**B**) Comparison of performance and energy consumption of one equivalent segment and four segments in bending motion. See fig. S6 for additional analyses. (**C**) Comparison of performance and energy consumption of one equivalent segment and four segments in circling trajectory following. See fig. S8 for additional analyses. (**D**) Strategy to save the energy consumption by employing lowest sufficient DOF for the task.

Because WBJs actively modulate only the electrical potential along the SMA actuator, the segmented regions remain intrinsically connected, allowing a single SMA actuator to be selectively actuated while dynamically segmented. When a WBJ is disconnected from the potential control circuit, it effectively becomes inactive, entering a floating state that passively adjusts the local electrical potential. Consequently, the active WBJs determine both the number and the effective length of the equivalent segments within the original single SMA actuator (Fig. 4A). By selectively activating WBJs and modulating their input signals, a WBJ–SMA actuated soft robot can dynamically adjust its effective DOF. As a result, the actuation space of the soft robot becomes much larger.

We further evaluate the performance under different numbers of equivalent segments within the expanded actuation space. A 260-mm-long soft robotic arm equipped with four WBJ–SMA actuators was evaluated using a step-tracking bending task (fig. S5), achieving a total bending angle of 90° with 10° increments and decrements under different equivalent segment configurations (Fig. 4B, fig. S6, and movie S4). Under closed-loop control, the arm exhibited comparable tracking performance across configurations, with average steady-state errors of 0.61°, 0.26°, 0.52°, and 0.76° for equivalent segment numbers ranging from one to four, respectively. The corresponding settling times to reach steady bending at each target angle were 2.17 s, 3.57 s, 3.36 s, and 3.08 s. Across the 18 step-tracking trials for each configuration, the maximum overshoot values were 1.35°, 0.83°, 1.58°, and 1.66°. These overshoots occurred predominantly when the arm was commanded to return to 80° after reaching the maximum bending angle of 90°, with noticeable oscillations observed during the return motion (movie S4). Reducing the power input mitigated this phenomenon, decreasing the average overshoot by more than 30%. However, this improvement came at the expense of slower dynamic performance, resulting in longer rise and settling times, as well as reduced steady-state accuracy (fig. S7). During the experiments, the main circuit current was recorded, with peak values of 1.36 A, 1.85 A, 2.10 A, and 2.76 A for one to four equivalent segments. By integrating the product of current and time and multiplying by the supply voltage, the energy consumption for each test was calculated, yielding values of 3.5 W, 4.1 W, 5.3 W, and 6.3 W as the number of equivalent segments increased.

Due to gravitational loading, the SMA actuator experienced uneven stress distribution, causing the robotic arm to fail to return to a straight configuration when actuated with a single equivalent segment (Fig. 4B). To quantify this limitation, we commanded the robotic arm to bend and rotate along a circular trajectory while recording the end-effector position (Fig. 4C, fig. S8, and movie S5). When only orientation feedback was used in the closed-loop controller, the resulting root mean square position errors (RMSE) differed substantially, measuring 51.79 mm, 10.70 mm, 8.14 mm, and 11.22 mm for one to four equivalent segments, respectively. By contrast, the average cumulative errors of the control parameters across the four actuation configurations were comparable, with values of 1.81 mm, 1.65 mm, 1.85 mm, and 2.36 mm, respectively (fig. S8). Since the length of the robot is too long, the single equivalent segment cannot overcome the environmental disturbance, resulting in the model does not work. Except the single case, further equivalent segments improve the dexterity of the robot, while there are no significant control accuracy improvement and the energy consumption increases.

Accordingly, pursuing excessively high degrees of freedom (DOF) is not always optimal. Because lower DOF configurations are generally more energy efficient, an alternative strategy is to adapt the robot’s effective DOF to task requirements. To demonstrate this capability, we tasked the robotic arm with delivering a magnet mounted at its tip to a target location to activate an LED (fig. S9). By progressively increasing the number of equivalent segments, the robot successfully avoided three successively introduced obstacles that obstructed the trajectory of the previous successful attempt (Fig. 4D and movie S6).

### Curve-based shape reconfiguration

Increasing the number of WBJs allows a single SMA actuator to be divided into an arbitrarily large number of effective segments, while in practice, a minimum spacing between WBJs is required to ensure reliable and independent actuation of each subsegment. Nevertheless, the introduction of WBJs enables levels of dexterity in soft robotic systems that are difficult to achieve using conventional actuation approaches. To demonstrate this capability, we constructed a planar soft robotic arm driven by two WBJ–SMA actuators, with eight WBJs on each, and programmed it to follow user-defined planar curves specified through an interactive interface (movie S7).

**Fig. 5.**
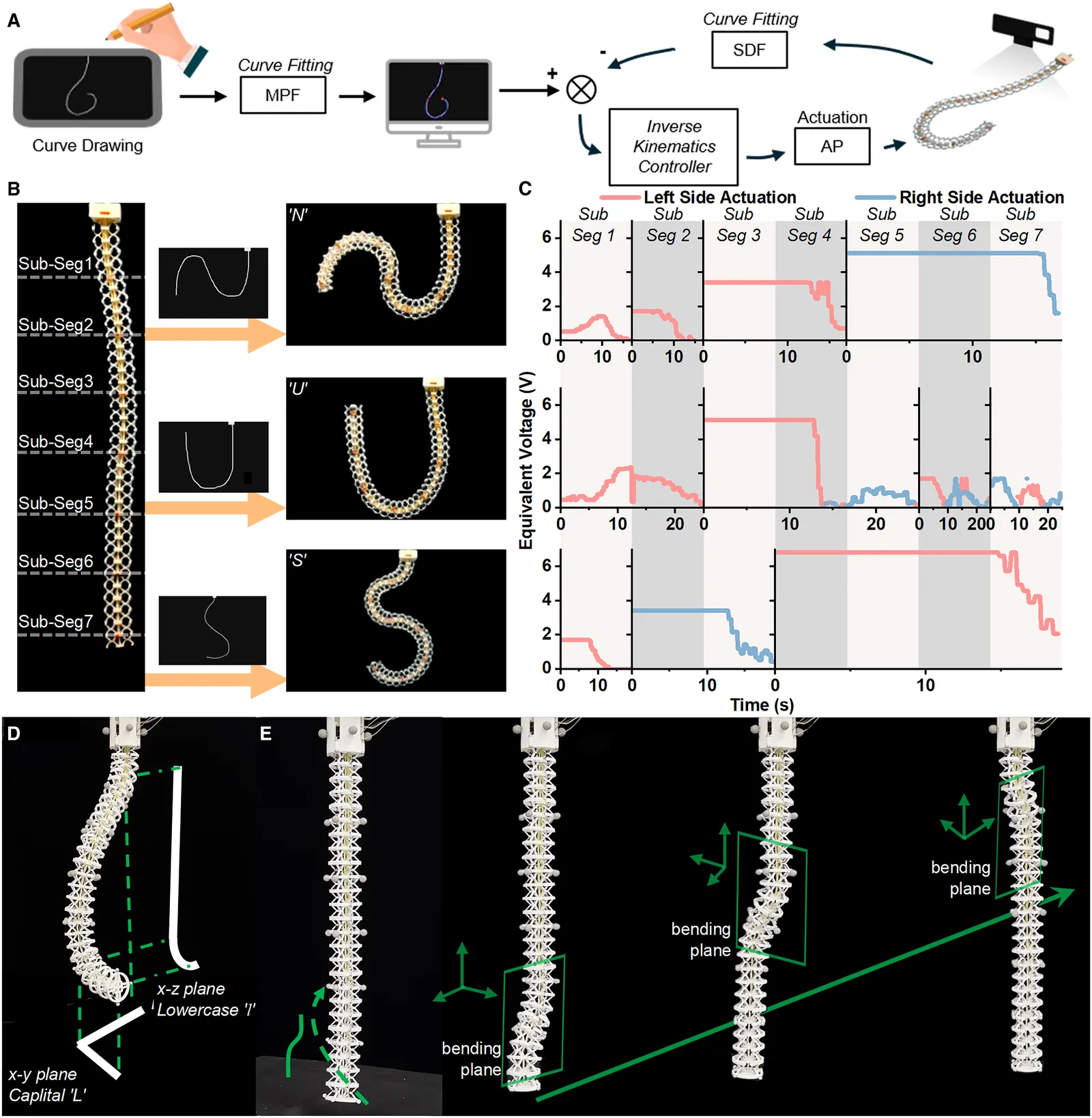
Highly dexterous motion achieved by the WBJ actuation. (**A**). The workflow of the shape reconfiguration of a soft robot following the drawn curve. (**B**) Design of the seven sub-segments robot and its performance on the letter following. (See fig. S10 for additional illustration). (**C**) The actuation of different equivalent segments during the curve following. (**D**) The spatial dexterous deformation of the WBJ-SMA robotic arm. (**E**) Spatial deformation of a helical propagating wave in the robotic arm.

To minimize energy consumption by using the lowest sufficient DOF, the target curve was first approximated using minimum piecewise fitting (MPF), which reduces the number of equivalent segments required to reproduce the desired shape. Closed-loop control was then implemented to track the resulting segmented target curve using visual feedback from a camera (Fig. 5A). Because one end of the robotic arm was fixed, the target curve was constrained to begin in the vertical direction. In the experiments, the arm was tasked with tracing three letters, ‘N’, ‘U’, and ‘S’ (Fig. 5B). Based on the MPF results, the corresponding actuation configurations, expressed as the number of subsegments activated along the arm, were 1–1–2–3, 2–3–1–1, and 1–2–4 for the three letters, respectively (fig. S10). The actuation voltage signals were subsequently modulated by both the closed-loop feedback controller and the lengths of the equivalent segments (Fig. 5C).

Beyond planar curve reconfiguration, the WBJ strategy also enables dexterous three-dimensional deformations. To demonstrate this capability, a robotic arm consisting of six reconfigurable subsegments was evaluated in three representative tasks (movie S12). In the first task, the arm formed a three-dimensional configuration whose projections onto the *x*–*z* and *x*–*y* planes corresponded to a lowercase “l” and an uppercase “L”, respectively (Fig. 5D). The remaining tasks further demonstrate dynamic spatial deformations, including the generation of a helical traveling wave through sequential bending in different planes while continuously reconfiguring the equivalent actuator segments (Fig. 5E and fig. S11), and sequential swinging motions achieved by dynamically subdividing and reconfiguring the equivalent segments (fig. S12).

### Multi-purpose actuation and drone deployment

Unlike vertebrates, whose upper limbs are functionally differentiated into the arm, wrist, and hand, the octopus arm lacks clear functional segmentation. Instead, the same musculature supports both locomotion and manipulation along the entire arm (Fig. 1A). In robotics, this corresponds to actuating both the robot body and the end effector using a unified actuation system rather than distinct actuators. To demonstrate that WBJ–SMA actuation can achieve similar functionality, we integrated the actuation of two end effectors with two subsegments of the robot body and deployed the system using a drone to perform an environmental sampling task.

**Fig. 6.**
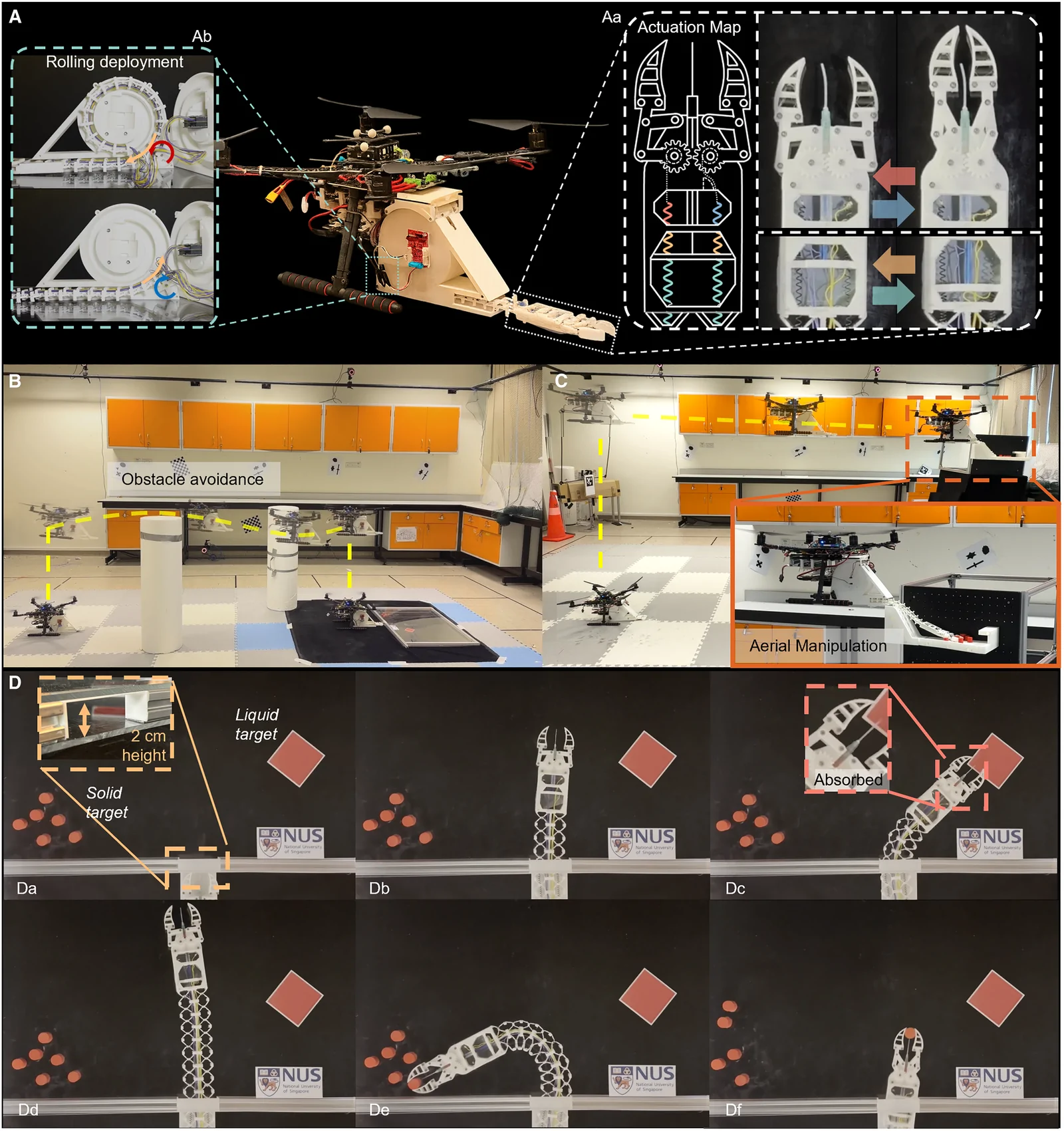
Multi-purpose actuation with WBJ actuation and its possible application. (**A**). Schematic of the drone deployed multi-purpose actuation system. (**Aa**) The actuation map of multiple end effectors by the same SMAs. (**Ab**) The rolling deployment for the exertion and retraction of the soft robot arm. (**B**) The deployment of the soft arm by drone and the obstacles avoidance. (**C**) Aerial manipulation of the soft arms into a height differences space. (**D**) The multi-phase sampling task in a confined space achieved by the multi-purpose actuation with WBJ.

Building on the planar robotic arm design, we integrated a multipurpose end effector capable of both grasping and syringe-based fluid sampling at the distal end of the arm (Fig. 6Aa). The ends of two SMA actuators were connected to gears via cables, enabling antagonistic actuation to open and close the gripper. The gripper achieved a maximum load capacity of 2.98 N while weighing only 31.2 g, corresponding to a load-to-weight ratio of 9.7 (fig. S13). In parallel, two WBJs were fixed to the end-effector frame, while intermediate WBJs were attached to a sliding pad connected to the syringe plunger. Actuation of the upper and lower subsegments translated the pad linearly, thereby enabling controlled liquid aspiration and ejection (movie S8). Meanwhile, with the anisotropic mechanical structure and the inherent compliance of the SMA actuators, the planar robot can be passively rolled for compact storage (Fig. 6Ab). By rotating a paddle to push and pull the segmented structure, the robot arm can also be actively deployed and retracted in a controlled manner (movie S9).

By enabling multiple actuation functions to be integrated into a single continuous actuator, the WBJ strategy minimizes the number of actuators, wiring, and transmission components required for the robotic system. Owing to this compact architecture, together with the lightweight SMA actuators and the compact actuation board we developed (fig. S14), the robotic arm itself weighs less than 130 g, with a total system mass of approximately 450 g including all auxiliary components. This mass falls within the payload capacity of most commercially available drones, enabling aerial deployment. To demonstrate this capability, we mounted the system on a custom-built drone (Fig. 6A) and successfully navigated the drone through obstacles to deploy the robot into a target area assumed to be difficult to access such as a rooftop (Fig. 6B, figs. S15 and S16, and movie S10). We then conducted test experiments involving the collection of both liquid and solid environmental samples in a confined space. Despite an entry height of only 2 cm, the robot was able to insert into the space and successfully collect liquid and solid samples through syringe-based absorption and grasping, respectively (Fig. 6D and movie S11). In addition to confined-space sampling, an in-air manipulation experiment was conducted. While suspended by the drone, the robotic arm successfully reached across different heights to collect objects (Fig. 6C and movie S13).

## DISCUSSION

In contrast to vertebrate muscles, which are organized primarily in parallel to generate large forces, octopus muscles are arranged in series to enhance maneuverability. Inspired by this distinctive neuromuscular architecture, we introduce a WBJ-enabled actuation strategy as a complement to advances in actuator technologies. SMA is employed here as a model actuator to demonstrate the concept, in which a single continuous actuator is segmented into multiple independently addressable functional units while preserving electrical and mechanical continuity. WBJs are simple to fabricate and integrate, enable rapid and robust assembly, and localized actuation within a single continuous actuator. As a result, a single actuator can generate high-DOF motion, allowing soft robots based on this architecture to perform behaviors that are difficult to achieve with conventional actuation strategies (*38*–*40*), including active variation of effective DOF, tracking of complex planar curves, and actuating multiple end effectors using the same actuator.

Conventional SMA-actuated soft robots, particularly those with large DOF, rely on bulky electronics or power supplies to accommodate the accumulated current demand of multiple parallel actuators (*41*–*43*). In such systems, fabrication variability leads to nonuniform current distribution, local overheating, and accelerated fatigue failure. By contrast, the WBJ approach segments an intrinsically single actuator rather than assembling many separate ones, thereby enabling seamless electrical continuity across segments and promoting uniform actuation with reduced current demand. Beyond improved electrical robustness, kinematic analysis predicts that the WBJ architecture provides more than a twofold increase in reachable workspace and approximately a 1.5-fold increase in manipulability compared with conventional stacked actuator designs of the same robotic length. In practice, this advantage is even more pronounced. Experimental comparisons under both equal robotic length and equal total actuator length consistently favored the WBJ architecture, as fabrication-induced variability prevents stacked configurations from fully realizing their theoretical kinematic performance, whereas the WBJ architecture maintains more uniform electrothermal actuation and therefore operates much closer to its theoretical limit. Heat conduction from locally actuated regions to adjacent segments influences overall actuator performance, this thermal cross-talk could be mitigated through increasing actuation speed, for example, increasing the actuation current from 1 A to 2 A and reducing the heating time reduced the unintended over-contraction from 12.3 mm to 1.97 mm.

The WBJ approach also reveal the requirements of bulky control systems and substantial computational resources in actively controlling the large amount of DOFs in soft robots (*44*, *45*) . By actively modifying the robot’s effective segmentation through floating WBJs, thereby providing the minimum sufficient DOFs required to accomplish a given task, our results demonstrate that control accuracy improved by 78% at the highest equivalent DOFs, while energy consumption was reduced by 44% at the lowest case. Although demonstrated here using SMA because of its intrinsic electrical conductivity, the proposed strategy can be extended to other continuum actuators, particularly thermally responsive systems, provided that localized activation can be achieved through appropriate electrical routing.

Combined with a simple fabrication process, the proposed WBJ-based reconfigurable-DOF strategy enables rapid construction of slender robotic arms with up to 14 planar and 24 spatial actuation DOFs within hours. The resulting system successfully demonstrated a more complex shape-following task when comparing to existing shape-control approaches that focus primarily on simple curves (*46*–*48*). Furthermore, following the octopus behavior, we employ the same SMA actuators to drive both the robot body and two different end effectors. Since no additional actuation components are required beyond the electronics, the 0.6 m-length manipulator-gripper system could be driven by a compact actuation board (40 mm in diameter). The reduced energy consumption, high dexterity, and compact architecture enabled by the WBJ strategy substantially broaden the deployment capability of the robotic system. Combined with its compliant, lightweight structure, this design enables the robot to be rolled into a small enclosure, transported by a standard drone, deployed into confined spaces, perform aerial manipulation across height differences, and used to manipulate both liquid and solid samples.

Despite these advantages, the relatively low operating frequency of SMA actuators remains a key limitation. A cooling phase will be required to accelerate the actuator’s recovery to its initial state. With the air-cooling strategy adopted in our design, this recovery process typically takes several seconds. As a result, the controller must carefully limit overshoot within the feedback loop, which constrains both convergence speed and control accuracy. Incomplete cooling of the SMA actuator causes it to exhibit spring-like behavior, resulting in oscillations that are further amplified by the slender geometry of the robotic arm. This limitation becomes particularly evident during reverse motions, such as returning from a bending angle of 90° to 80°. Although the antagonistic actuator configuration partially mitigates this effect, the slow thermal response hampers accurate repositioning, and simultaneous force generation by opposing actuators can induce buckling or failure of the central backbone. Future integration of active cooling system (*49*, *50*) or taking advance of environmental cooling capability (*51*) would make up for the current shortcomings. Increasing the actuation frequency would substantially enhance the controllability of WBJ–SMA systems, thereby improving performance and expanding the applicability of this approach to hyper-redundant robotic platforms such as soft robots.

## MATERIALS AND METHODS

### Fabrication and removement of the WBJ

Commercial SMA spring actuators were used in this study, with a wire diameter of 0.02 inch and an outer spring diameter of 0.136 inch (Dynalloy Inc., USA). The complete fabrication process is shown in movie S1. Electrical wires were stripped and tightly wound around designated locations on the SMA actuator to ensure reliable electrical contact. A two-part conductive epoxy adhesive (529, Ausbond Co. Ltd., China) was then mixed and applied to encapsulate each wire-bonding junction (WBJ), serving as a protective sheath that ensures mechanical stability, provides interlocking to anchor the actuator within the robot body, and offers redundant electrical connectivity in the event of wire failure. The adhesive was cured at room temperature for 12 h to complete fabrication.

Because the cured conductive epoxy is more brittle than the SMA substrate, the WBJ sheath can be readily removed by making a small incision with a flat-blade shear, exposing the underlying wound wire and allowing it to be detached as needed.

### WBJ characterization experiments

Two sets of samples were prepared for WBJ characterization experiments. The first set consisted of SMA actuators with 10 coils and WBJs bonded at both ends, representing individual actuators. The second set consisted of SMA actuators with 15 coils, with WBJs bonded at both ends and an additional WBJ bonded at the 10th coil, representing WBJ-segmented actuators. Cable knots were formed at the ends of all SMA samples to facilitate fixation during testing.

For stroke measurements, payloads of 50 g, 100 g, 150 g, and 200 g weight were suspended from the tested actuator (fig. S1A). A laser distance sensor (BOJKE BGL-235NMZ, Guangdong Boyijingke Sensing Co., Ltd., China) was positioned below the actuator and connected to a data acquisition card to record the vertical displacement of the payload. Prior to each test, all SMA samples were pre-stretched using a 200 g load to ensure consistent initial conditions. Actuation current was supplied and regulated using an adjustable bench power supply.

Output force measurements were conducted using the same SMA samples, arranged horizontally to eliminate gravitational effects. A force meter equipped with a hook was used to measure the generated force. During testing, each sample was pre-stretched to 100%, 200%, and 300% of its fully contracted length using a linear stage coupled to the force meter. To minimize the influence of residual stress within the actuator, an additional 2 mm stretch–release cycle was applied before each measurement to reset the actuator to the target position.

Tensile testing was performed using an additional set of samples, each containing a single WBJ positioned at the midpoint of the SMA actuator. The samples were mounted in a universal testing machine (34TM-5, Instron Inc., USA), with the SMA wire and bonded electrical wires clamped at opposite ends. Tensile loading was applied at constant extension rates of 15 mm/min and 30 mm/min until failure of the WBJ occurred.

### Soft robot design for embedding WBJs

All soft robotic systems presented in this work follow the same modular design. The basic structural unit is a rhombus-shaped module with 3-mm-thick top and bottom platforms that anchor the SMA actuator. All robot body are 3D-printed by Thermoplastic Polyurethane (TPU). By tuning the geometric parameters of the module, the compliant structure provides sufficient restoring force to elongate the SMA actuator after contraction. Each unit has a height of 20 mm. In the compact arrangement, adjacent units share the top and bottom platforms, forming a mechanically continuous structure. In contrast, the stacked arrangement consists of mechanically independent units separated by 2-mm gaps to provide electrical isolation and accommodate cable routing. The terminal units at both ends are truncated to half height, forming stable triangular structures for fixation. As a result, for a total structure height of 100 mm, the compact arrangement accommodates four active units per chain, whereas the stacked arrangement supports only three active units within the same length. Similarly, the robot arm used for bending and circular trajectory following task is with 12 active units per chain, with 3 units per sub-segment for actuation. At the center, there is a cylinder with diameter of 2.5 mm works as the backbone.

Instead of employing four chains to form a three-dimensional structure, the planar robot uses two chains positioned on the left and right sides. The curve-following arm consists of 35 units, organized into subsegments of five units each. The drone-deployable arm comprises 22 units, of which the first 16 units are embedded with WBJ–SMA actuators, forming four subsegments of equal length.

### Manipulability and workspace analysis between different configurations

Based on the derived kinematics of the segmented continuum robot (note S1 and fig. S17), the transformation matrices of two configurations are

$$\begin{array}{c} T_{\text{Compact}}=T\left(L_{𝒷}\right)T_{1}T_{2}T_{3}T_{4}T\left(L_{𝓉}\right)\\ T_{\text{Stack}}=T\left(L_{𝒷}\right)T\left(L_{𝒸}\right)T_{1}T\left(L_{𝒸}\right)T_{2}T\left(L_{𝒸}\right)T_{3}T\left(L_{𝒸}\right)T\left(L_{𝓉}\right) \end{array}$$ (1)

where T(L) is the translation matrix with a translation along z-axis for L. $L_{𝒷},L_{𝓉}$ and $L_{𝒸}$ are the length between the base and the first active segment, the robot tip and the top of the last active segment, and the connector length, respectively. Accordingly, the Jacobian matrices of two configurations are

$$\begin{array}{c} J_{\text{Compact}}=\left[Ad_{T\left(L_{𝒷}\right)}J_{1}\;Ad_{T\left(L_{𝒷}\right)T_{1}}J_{2}\;\cdots \right]\\ J_{\text{Compact}}=\left[Ad_{T\left(L_{𝒷}\right)T\left(L_{𝒸}\right\}}J_{1}\;Ad_{T\left(L_{𝒷}\right)T\left(L_{𝒸}\right)T_{1T}\left(L_{𝒸}\right)}J_{2}\;\cdots \right] \end{array}$$ (2)

Then, the length of three axes of the manipulability ellipsoid is

$$\lambda_{\nu }=\mathrm{eig}\left(J_{\nu }J_{\nu }^{T}\right)\;\;\lambda_{\omega }=eig\left(J_{\omega }J_{\omega }^{T}\right)$$ (3)

and the ratio for expressing the manipulability at each point of the workspace are

$$\epsilon_{\nu }=\frac{\text{min}\left(\lambda_{\nu }\right)}{\text{max}\left(\lambda_{\nu }\right)}\;\;\epsilon_{\omega }=\frac{\text{min}\left(\lambda_{\omega }\right)}{\text{max}\left(\lambda_{\omega }\right)}$$ (4)

### Bending and circling experiment of the robot arm

For closed-loop control, four inertial measurement units (IMUs; JY61P, WitMotion, Shenzhen, China) were embedded within the robot arm, positioned near the distal end of each equivalent subsegment. Sensor data were used selectively, such that only the IMUs adjacent to activated WBJs contributed to the control algorithm. Consequently, the number of active sensors always matched the number of equivalent segments. Each equivalent segment was controlled independently, with its target bending angle determined by the segment’s length fraction relative to the total arm length. Control was based on a piecewise constant curvature (PCC) model, combined with an improved state parameterization derived from the differential displacement between antagonistic actuator pairs, as proposed in prior work (*52*). A tuned PID controller was implemented to minimize tracking error by modulating the duty cycle of the PWM actuation signal.

During the bending experiments, each step was executed over a duration of 6 s. To quantify energy consumption, a current sensor was integrated into the power supply circuit, and the time-varying current was recorded. Power consumption was calculated by integrating the product of the measured current, actuation voltage, and time step, and then normalizing by the total measurement duration.

For the circling experiments, the controlled variables were the postures of the individual segments. An initial stabilization period of 20 s was applied, after which the trajectory was divided into bending and circling phases. The robot first executed a 60° bending motion using 10 discrete steps, each with a duration of 2 s. This was followed by the circling phase, which consisted of 40 steps, also executed with 2 s per step. A motion capture system (OptiTrack, NaturalPoint Inc., USA) was used to record the position of the end plate. To enable comparison between the experimental data and the desired trajectory, the recorded data were resampled based on timestamps and performance was evaluated by computing the RMSE.

### Electronics for the WBJ actuation

Because WBJ operation is based on assigning different electrical potentials along the actuator, the primary function of the electronic system is to connect each WBJ to appropriate voltage sources and to modulate these connections using pulse-width modulation (PWM) control signals. At each WBJ connection node in the circuit (fig. S14B), a P-channel MOSFET and an N-channel MOSFET are placed on opposite sides, connecting the node to the supply voltage and ground, respectively.

Voltage-divider resistors are used to ensure reliable MOSFET switching, while a transistor stage is employed to transfer control signals and switch operating modes. To support scalable actuation, a compact control board was developed that accommodates up to 38 WBJ channels (fig. S14A). An Arduino Mega 2560 microcontroller is mounted on the underside of the board, with each general-purpose input/output (GPIO) pin assigned to control an individual MOSFET channel.

### Communication protocol of the WBJ-SMA actuation

To enable asynchronous actuation of different WBJ-segmented regions along the SMA actuator, we designed a dedicated communication and control protocol. The low-level actuation control is executed on the embedded microcontroller by directly manipulating GPIO registers. Communication between the embedded Arduino and the host computer is implemented via an I2C interface. A translation layer was implemented between the high-level controller and the embedded Arduino to map control commands to hardware-level operations. To prevent unintended potential differences between WBJs arising from simultaneous switching, equivalent segments were actuated sequentially rather than in parallel. Specifically, each subsegment of the target SMA region was actuated in order within a fixed time window. Short idle intervals were inserted after each PWM actuation cycle to allow communication, sensor data acquisition, and control updates.

Figure S18 illustrates an example actuation sequence used in the curve-following experiments. Each subsegment was assigned a base actuation interval of 1 ms, and each equivalent segment was actuated according to the number of its constituent subsegments and the specified PWM duty cycle. Owing to hardware constraints, the minimum controllable actuation interval for a single subsegment was limited to 0.1 ms.

### Curve following experiment

During the experiments, the robot was labeled with visual markers at each subsegment and controlled using feedback provided by a camera-based tracking system. A graphical user interface (GUI) was developed to allow users to draw the desired target trajectory. The start point was constrained to the upper central region of the canvas, and the drawn curve was automatically checked to ensure a vertical initial direction and the absence of self-intersections.

Once the curve satisfied these requirements, a curve-fitting procedure termed minimum piecewise fitting (MPF) was applied. Specifically, the recorded curve was first rescaled to match the effective length of the robot arm in the camera frame. A curvature-based fitting algorithm was then used to approximate the target curve with the minimum number of smoothly connected circular arcs, each with a length constrained to integer multiples of the subsegment length in the image frame. If the fitting error for all candidate solutions exceeded a predefined threshold, a default configuration using seven subsegments was selected. The fitted curve was subsequently overlaid with the original drawing to allow user verification.

After confirmation, an inverse kinematics controller was used to generate actuation commands that drove the robot arm toward the target shape via the actuation protocol (AP). Depending on the number of active control segments, visual feedback was processed using selective dot fitting (SDF), in which only markers corresponding to the fitted segments were used to estimate the current arm configuration and provide feedback to the controller.

For spatial deformation, the target configuration was predefined by the bending angle and bending plane of each equivalent actuator segment. Four reflective markers were attached around each WBJ to define the local cross-sectional plane of the robotic arm. The three-dimensional positions of the markers were captured using a motion capture system (OptiTrack, NaturalPoint Inc., USA), from which the bending angle and plane of each segment were reconstructed. These measurements were fed back to the closed-loop controller to regulate the actuation of the corresponding equivalent segments.

### Multi-effector design

Multiple end effectors were integrated along the arm, including a gripper and a syringe-based sampler. The gripper consists of TPU-printed, fin-shaped fingers mounted on a PLA-printed frame and actuated through a four-bar linkage mechanism coupled with gears. A thin SMA wire was used as a cable to connect the ends of the SMA actuators to the two gears, enabling clockwise rotation of both gears and thereby antagonistically opening and closing the gripper.

The gripper frame was connected to an additional TPU-printed frame that interfaces with the arm body. Guide chutes were formed on both sides of this frame, with a sliding pad positioned centrally to translate along the frame. The sleeve of the syringe was placed along two frames and glued. The plunger of the syringe was bonded to the sliding pad, and two WBJs were introduced to connect the SMA actuator to the pad. Actuation of the corresponding SMA subsegments drove linear motion of the pad, enabling controlled aspiration and ejection of fluid.

### Drone deployment experiment and sampling task

A custom-developed drone was used for the deployment experiments. A dedicated connector was designed to mount the storage shell securely to the drone frame. During the experiment, the drone first took off from the ground and navigated toward the target location. Two tall foam cylinders were positioned between the start and end points to simulate obstacles along the deployment path. To accommodate these obstacles, the drone followed a predefined three-segment, piecewise-linear trajectory, after which it reoriented to face forward and landed at the target location. A motion capture system was used to record the position and orientation of the drone throughout the flight, with the resulting trajectory and posture data shown in figs. S15 and S16, respectively.

For the aerial manipulation experiment, the target object was positioned at a height of 1.6 m on a 3D-printed platform mounted to an optical breadboard, creating a vertical height difference of 10 cm. The robotic system was powered by the drone battery through a DC voltage regulator (DFR0205, DFRobot, China), which provided a regulated 12 V supply to the actuator and control electronics. An Arduino Uno was used to receive commands transmitted from a handheld joystick via Bluetooth (HM-10) and communicate with the custom-developed actuation control board. During the experiment, the drone was first flown to the target location, after which the robotic arm was remotely operated to perform object collection.

The sampling task experiment was conducted using a custom-built enclosure composed of two transparent acrylic plates supported by aluminum profiles (20 mm by 20 mm) along the perimeter. A narrow entry opening was positioned at the center of the enclosure, with a height of 20 mm and a width of 54 mm. Several 3D-printed cylinders were used as solid sampling targets, while red-dyed water served as the liquid sampling target. During the experiment, the robot arm was first inserted into the enclosure by rotating the insertion motor for three revolutions, corresponding to a forward translation of 12 structural units. The first four design units on the right side were then actuated to bend the arm toward the liquid target, after which the syringe end effector was activated to aspirate the liquid sample. The arm was subsequently returned to a straight configuration by actuating the left-side actuators. To reach solid targets located deeper within the enclosure, an additional rotation of the insertion motor was applied, followed by two successive actuations of the first two subsegments. Once the gripper was aligned with a solid target, it was activated to grasp the object. Finally, the antagonistic SMA segments were actuated to straighten the arm, and the entire system was retracted, successfully retrieving both liquid and solid samples.

## Supplementary Materials

### The PDF file includes:

Supplementary Materials Figs. S1 to S18 Legends for movies S1 to S13 References
