# Supplementary material for: Octopus-inspired muscle activation unlocks high-DOF motion from a single actuator
Source: Sci Adv. 2026 Sep 23;12(39):eaei4282. doi: 10.1126/sciadv.aei4282 (PMC13600154; doi:10.1126/sciadv.aei4282)
Supplement: Supplementary file 1 [file sciadv.aei4282_sm.pdf]

Science Advances
AAAS

Supplementary Materials for

Octopus-inspired muscle activation unlocks high-DOF motion from a single actuator

Wenci Xin *et al.*

Corresponding author: Email: Zhexin Xie, xiezx@sustech.edu.cn; Cecilia Laschi, mpeclc@nus.edu.sg

Sci. Adv. **12**, eaei4282 (2026)
DOI: 10.1126/sciadv.aei4282

The PDF file includes:

Supplementary Materials
Figs. S1 to S18
Legends for movies S1 to S13
References

Other Supplementary Material for this manuscript includes the following:

Movies S1 to S13

Supplementary Materials

Note S1. Kinematics of the segmented continuum soft robot

For each deformable segment, as illustrated in Fig. S17, since the backbone keeps a constant length and the actuators are symmetrical in pairs, such that:

$$\begin{aligned} l_1 + l_3 &= 2l \\ l_2 + l_4 &= 2l \end{aligned} \tag{1}$$

where l_1, l_2, l_3, l_4 are the length of actuators clockwise, and l is the constant length of the segment. For continuum robots, the configuration of each segment is determined by a group of three parameters (L, ϕ, θ), corresponding to the length, rotating angle, and the curvature angle, respectively. The length of the segment is constant l and the rest parameters are:

$$\phi = \arctan\frac{l_4 - l_2}{l_3 - l_1} \tag{2}$$

$$\theta = \frac{(l_1 + l_3 + l_4 - 3l_2)\sqrt{(l_4 - l_2)^2 + (l_3 - l_1)^2}}{4d(l_4 - l_2)} \tag{3}$$

where d is the distance between the actuator and the center, and θ will be calculated by replacing the l_2, l_4 by l_1, l_3 when l_2 and l_4 have the same length. Then, the transform matrix of each segment can be obtained by:

$$\mathrm{T} = \begin{bmatrix} \mathrm{R_z}(\phi) & 0 \\ 0 & 1 \end{bmatrix} \begin{bmatrix} \mathrm{R_y}(\theta) & \mathrm{l_y} \\ 0 & 1 \end{bmatrix} \begin{bmatrix} \mathrm{R_z}(-\phi) & 0 \\ 0 & 1 \end{bmatrix} \tag{4}$$

where

$$l_y = \begin{bmatrix} \frac{\mathrm{l}}{\theta}(1 - \cos\theta) & 0 & \frac{\mathrm{l}}{\theta}\sin\theta \end{bmatrix}^T$$

and $R_z()$ represents the rotation matrix around the z-axis. The tip position of the line segment can be obtained from the first three elements of the fourth column. By taking partial derivatives of these three positions with respect to the configuration parameters $[l, \phi, \theta]^T$, the linear velocity Jacobian matrix can be obtained:

$$J_v = \begin{bmatrix} -\frac{\mathrm{c}\phi(\mathrm{c}\theta - 1)}{\theta} & -\psi\mathrm{s}\phi\mathrm{s}\theta + \frac{\mathrm{l}\mathrm{s}\phi(\mathrm{c}\theta - 1)}{\theta} & \psi\mathrm{c}\phi\mathrm{c}\theta + \frac{\mathrm{l}\mathrm{c}\phi(\theta\mathrm{s}\theta + \mathrm{c}\theta - 1)}{\theta^2} \\ -\frac{\mathrm{s}\phi(\mathrm{c}\theta - 1)}{\theta} & -\psi\mathrm{c}\phi\mathrm{s}\theta - \frac{\mathrm{l}\mathrm{c}\phi(\mathrm{c}\theta - 1)}{\theta} & \frac{\mathrm{l}\mathrm{c}\phi(\theta\mathrm{s}\theta + \mathrm{c}\theta - 1)}{\theta^2} \\ \frac{\mathrm{s}\theta}{\theta} & 0 & -\psi\mathrm{s}\theta + \frac{\mathrm{l}(\theta\mathrm{c}\theta - \mathrm{s}\theta)}{\theta^2} \end{bmatrix} \tag{5}$$

where ψ refers to the extension of the tip and $c\theta$ means $\cos\theta$ for saving the space. From the physical meaning of Eq. 4, the angular velocity Jacobian matrix is:

$$J_\omega = \begin{bmatrix} 0 & -\cos\phi\sin\theta & -\sin\phi \\ 0 & -\sin\phi\cos\theta & \cos\phi \\ 0 & 1 - \cos\theta & 0 \end{bmatrix} \tag{6}$$

Finally, refer to (*53*) the Jacobian matrix of a multi-section continuum robot can be expressed as:

$$J = [\mathrm{J_0} \quad \mathrm{Ad_{T_0}J_1} \quad \mathrm{Ad_{T_{01}}J_2} \cdots] \tag{7}$$

where J_i is the Jacobian matrix of ith segment and $Ad_{T_{0m}}$ is the adjoint of the transformation matrix $T_{0m} = T_0 T_1 \ldots T_m$. For any transformation matrix T that is composed of rotation $\mathcal{R}$ and translation $\mathcal{P}$, its adjoint is:

$$\mathrm{Ad_T} = \begin{bmatrix} \mathcal{R} & [\mathcal{P}]_\times \mathcal{R} \\ \mathcal{R} & 0 \end{bmatrix} \tag{8}$$

with respect to the twist $[\nu, \omega]^T$, and $[\mathcal{P}]_\times$ indicates the skew-symmetric matrix of $\mathcal{P}$.

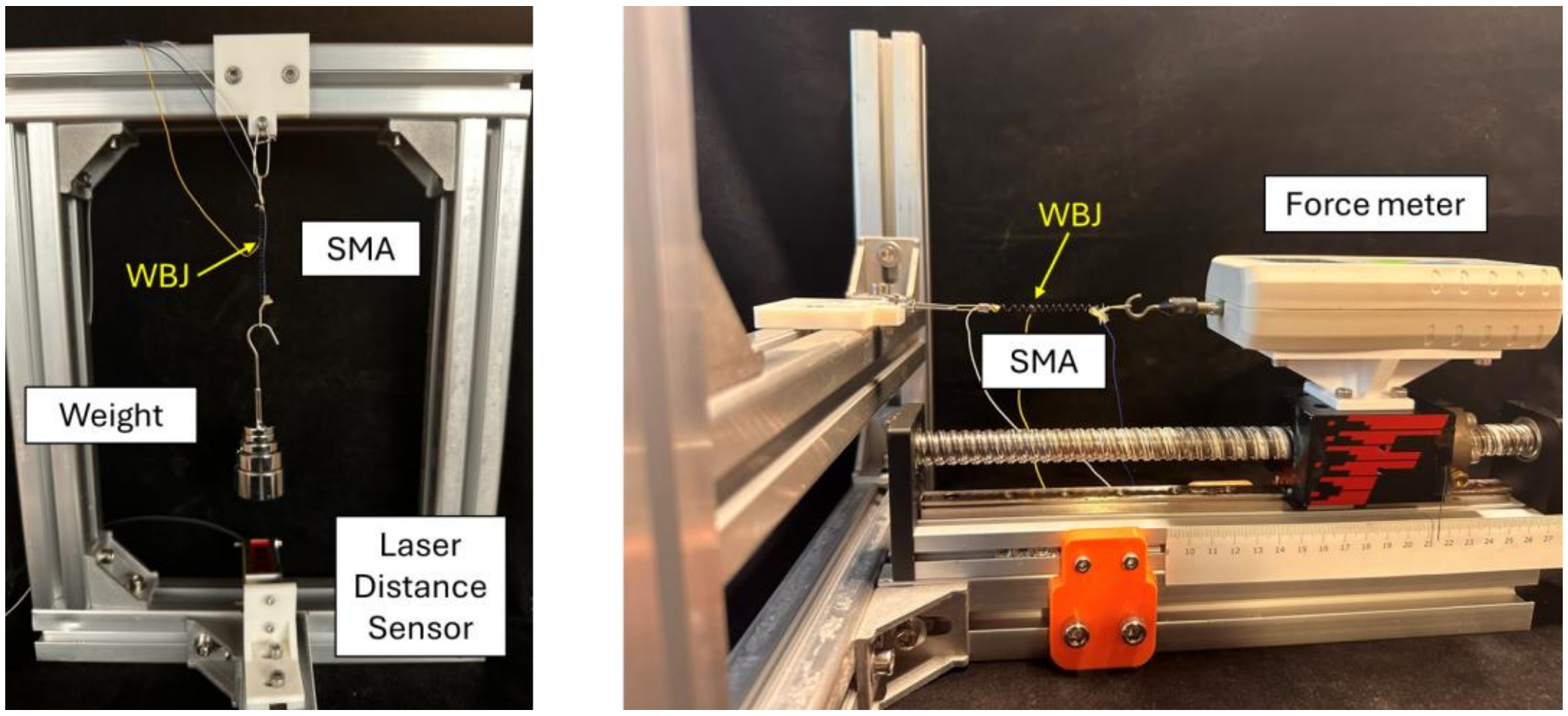

Fig. S1. Testing setup for the WBJ characterization

(Left) The stroke test of the WBJ-SMA under different loads. (Right) The output test of the WBJ-SMA under different elongating ratio.

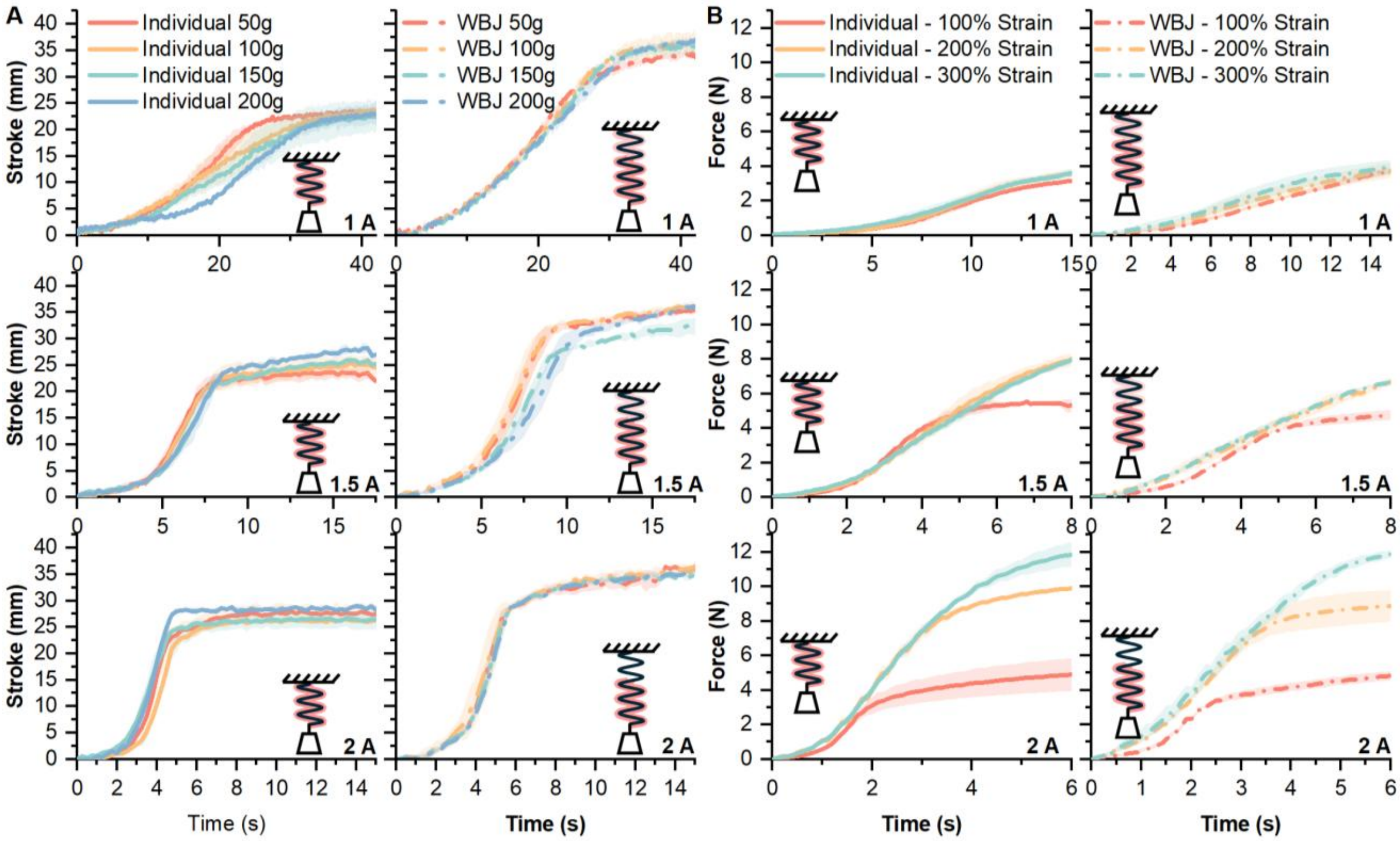

Fig. S2. Characterization of stroke and force under different current

(A) The performance of the stroke test under the current actuation on 1A, 1.5A and 2A. (B) The performance of the force output under the current actuation on 1A, 1.5A and 2A.

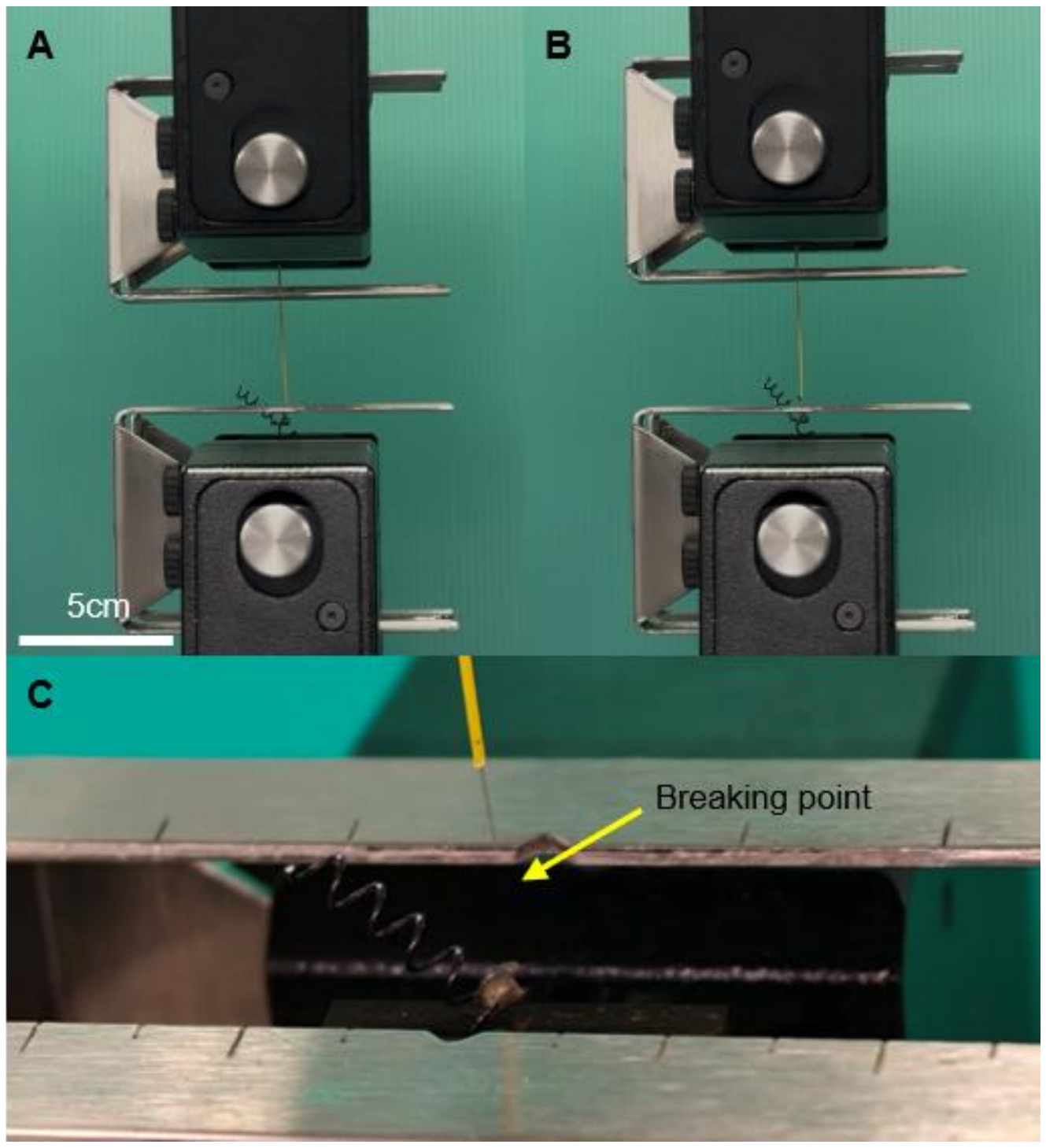

Fig. S3. Strength test of the WBJ actuator
(A) Experimental setup of the break strength test. (B) WBJ breaking moment during the test. (C) The breaking point of the WBJ.

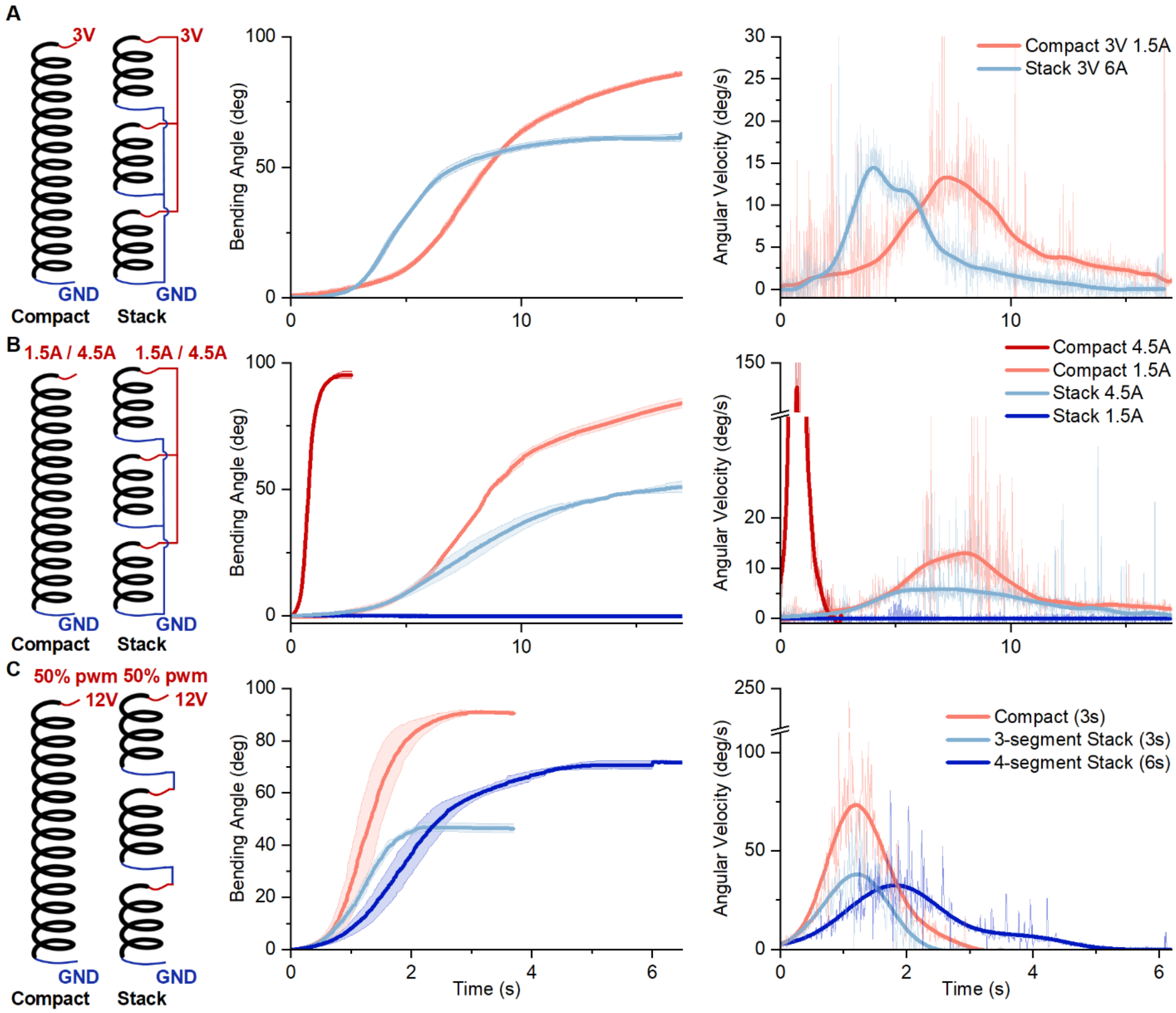

Fig. S4. Kinematic characterization of compact and stack arrangement under different actuating conditions

(A) Constant voltages with individual SMA actuators connected in parallel. (B) Constant current with individual SMA actuators connected in parallel. (C) PWM-controlled with individual SMA actuators connected in series. To ensure complete bending, the stacked configuration with the same robotic structural length (3-segment) was actuated for 3 s, whereas the configuration with the same total actuator length (4-segment) was actuated for 6 s.

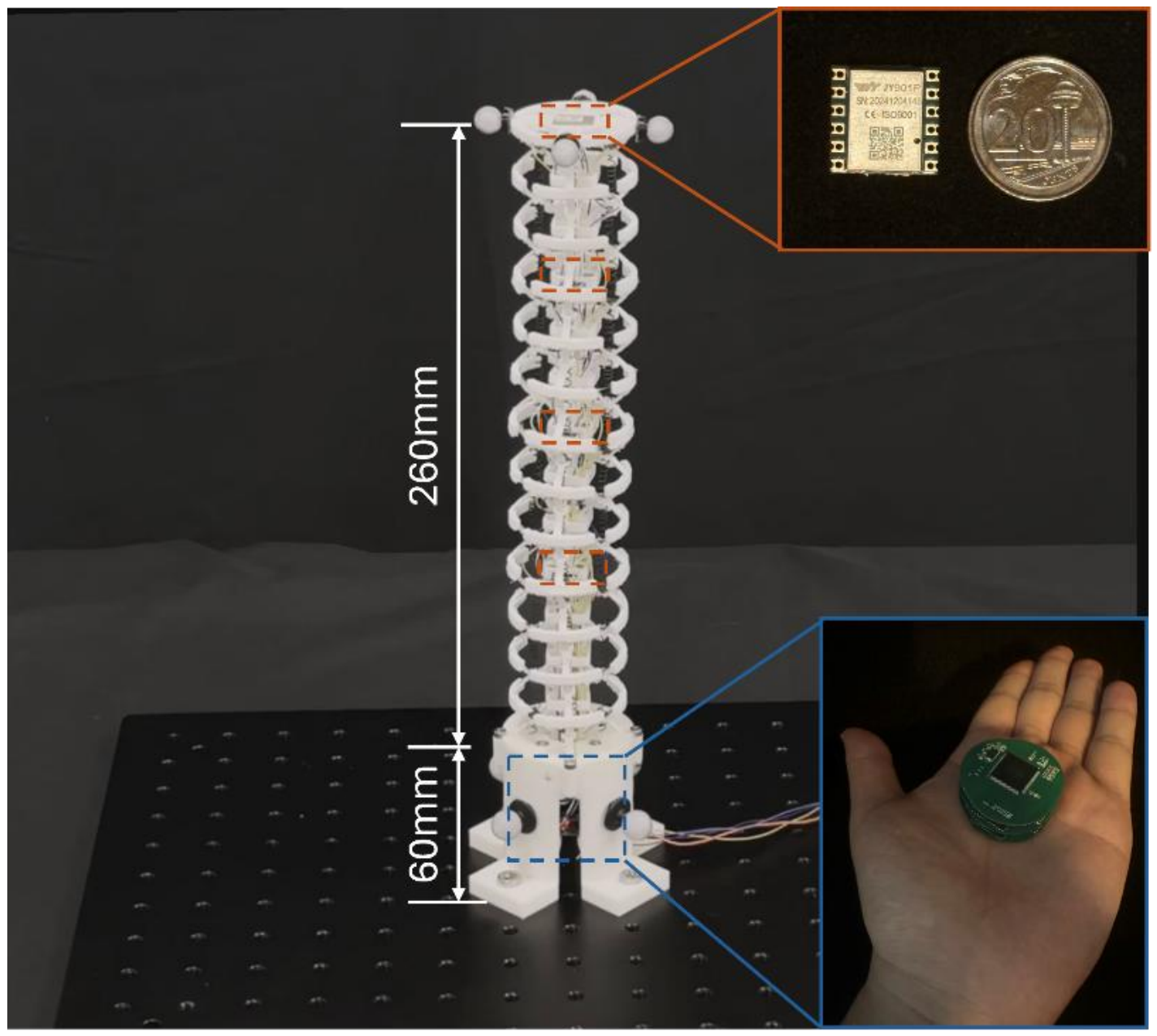

Fig. S5. System design of the variable DoF arm for bending and circling test

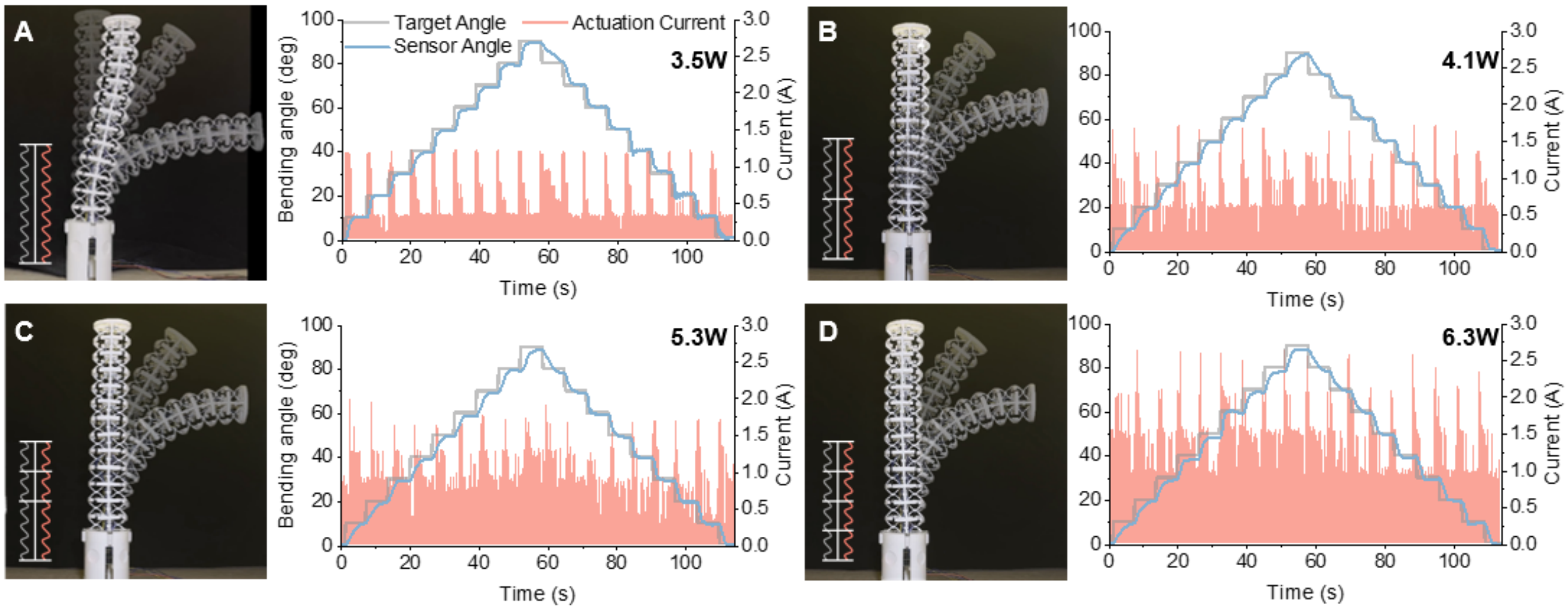

Fig. S6. Performance of the bending testing of the arm under different number of equivalent segments.
(A) One equivalent segment. (B) Two equivalent segments. (C) Three equivalent segments. (D) Four equivalent segments. This figure includes the same image shown in Figure 4B, reproduced here to facilitate comparison with the additional analyses.

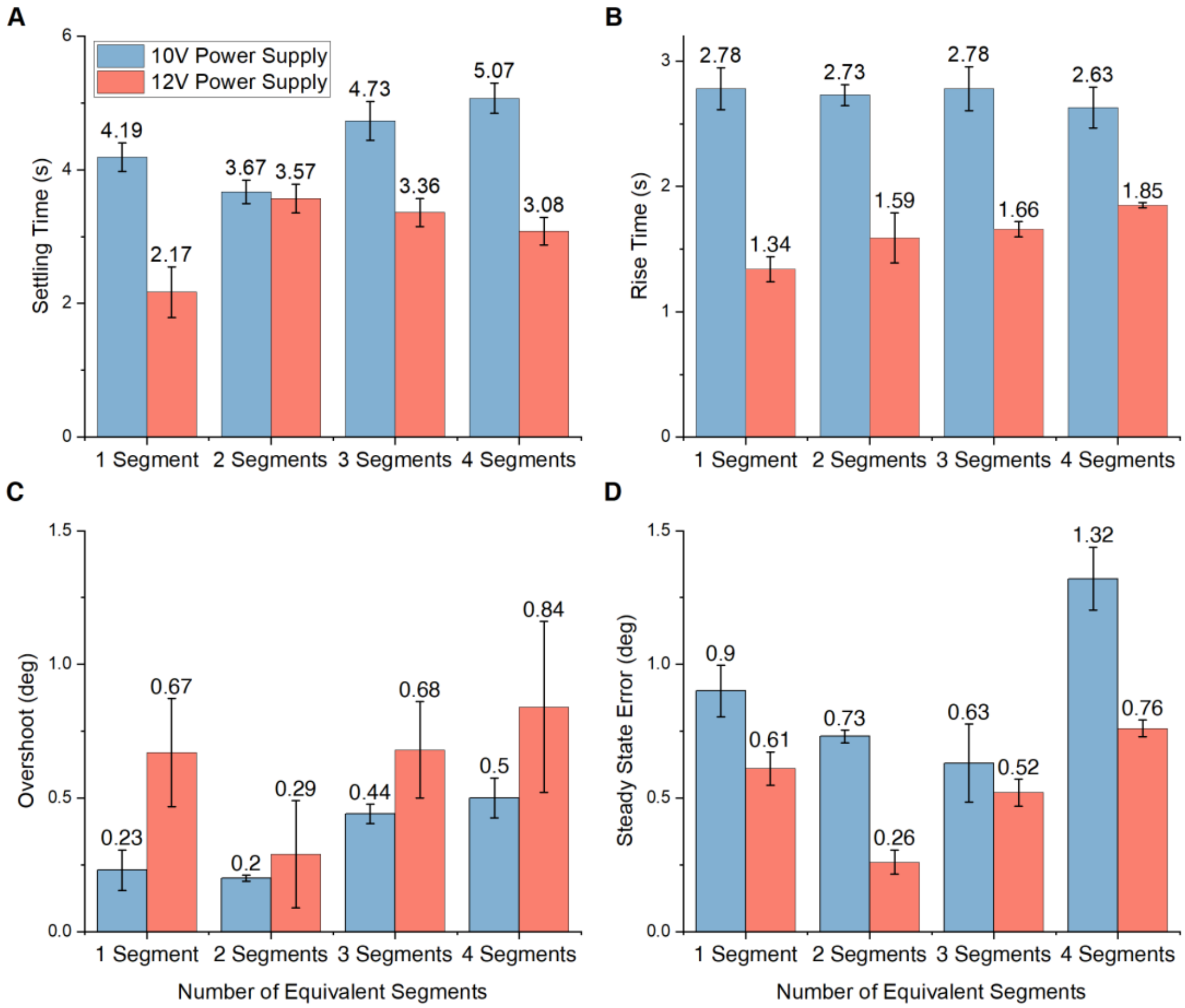

Fig. S7. Stability characterization of WBJ-SMA bending under different input power levels. (A) Settling time (time required for the response to remain within ±0.5° of the target angle). (B) Rise time (time required for the response to increase from 10% to 90% of the target angle). (C) Overshoot (maximum angular deviation beyond the target angle following each step input). (D) Steady-state error (angular error between the steady-state response and the target angle).

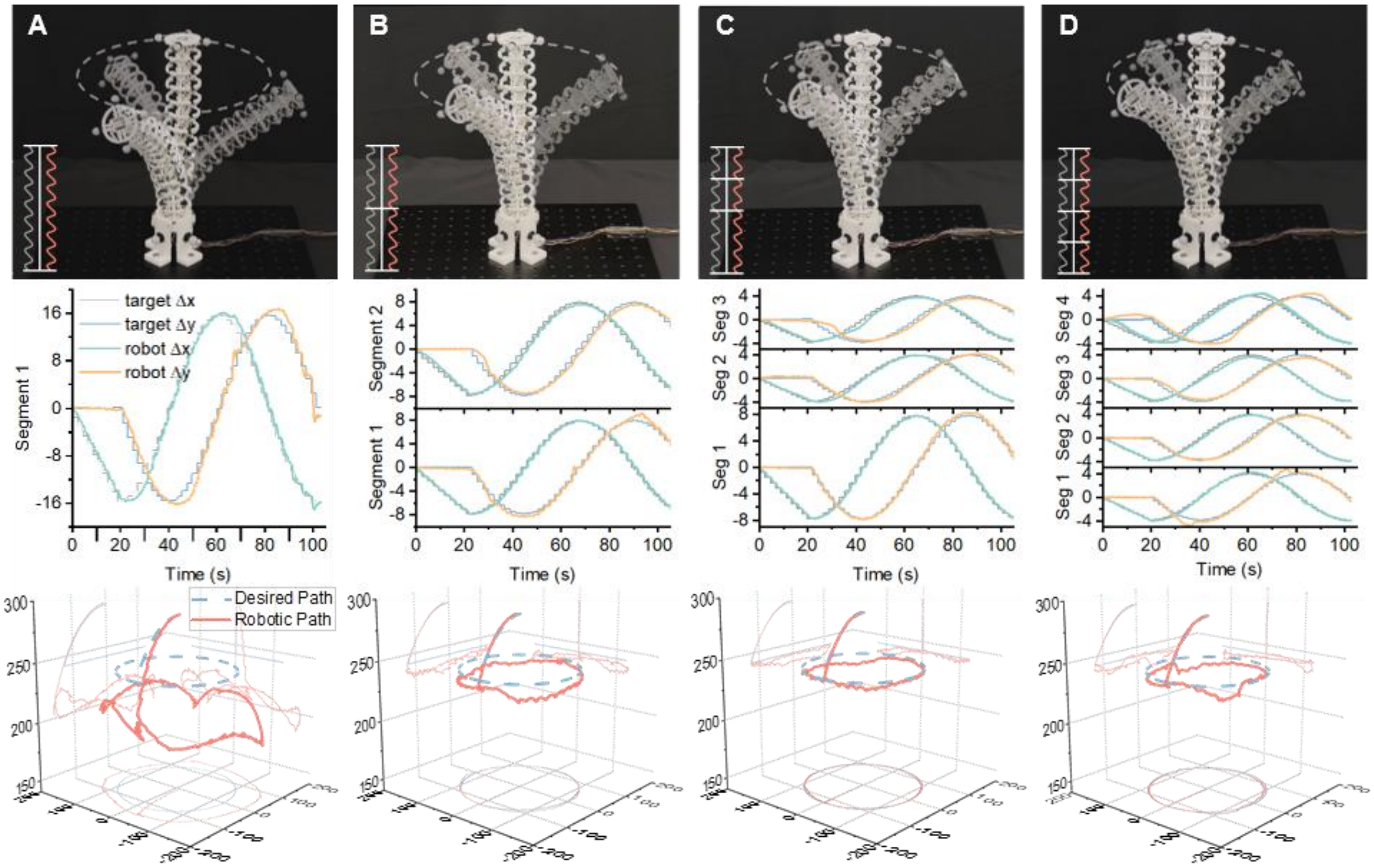

Fig. S8. Performance of the circular testing of the arm under different number of equivalent segments.

(A) One equivalent segment. (B) Two equivalent segments. (C) Three equivalent segments. (D) Four equivalent segments. This figure includes the same image shown in Figure 4C, reproduced here to facilitate comparison with the additional analyses.

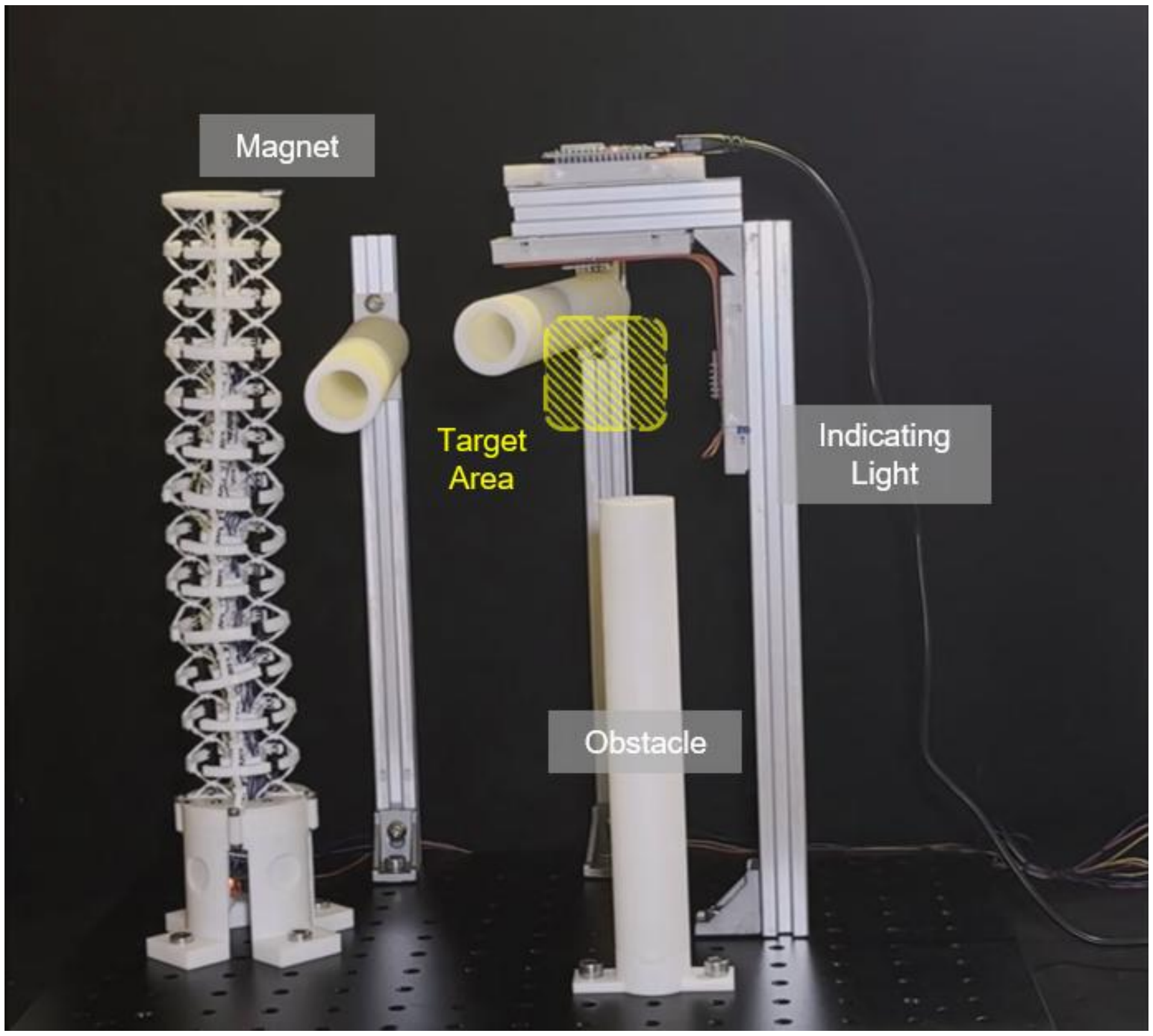

Fig. S9. Experimental setup for the obstacle experiment

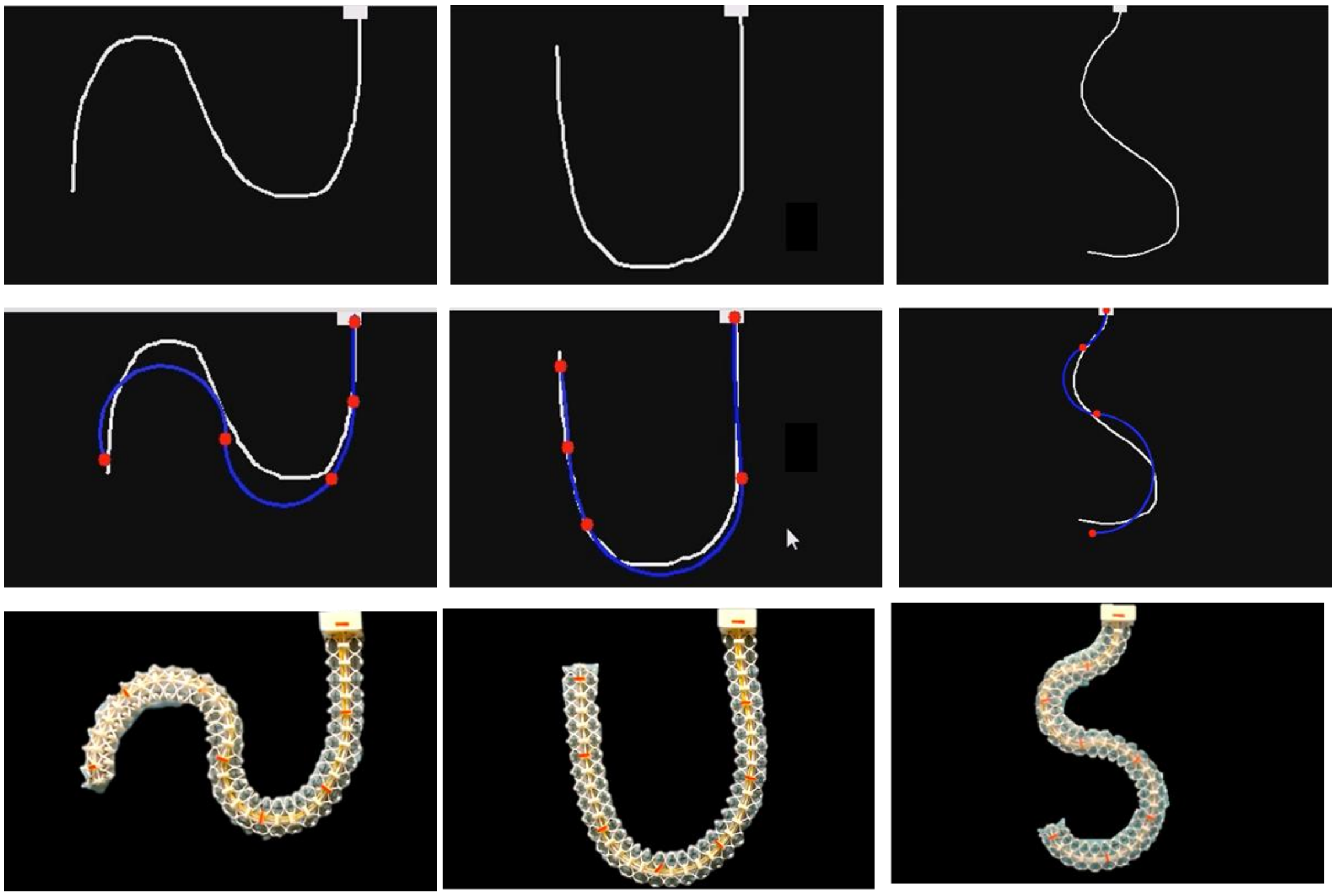

Fig. S10. Input, fitted and controlled shape for the letter 'N', 'U' and 'S'

This figure includes the same image shown in Figure 5B, reproduced here to facilitate illustration.

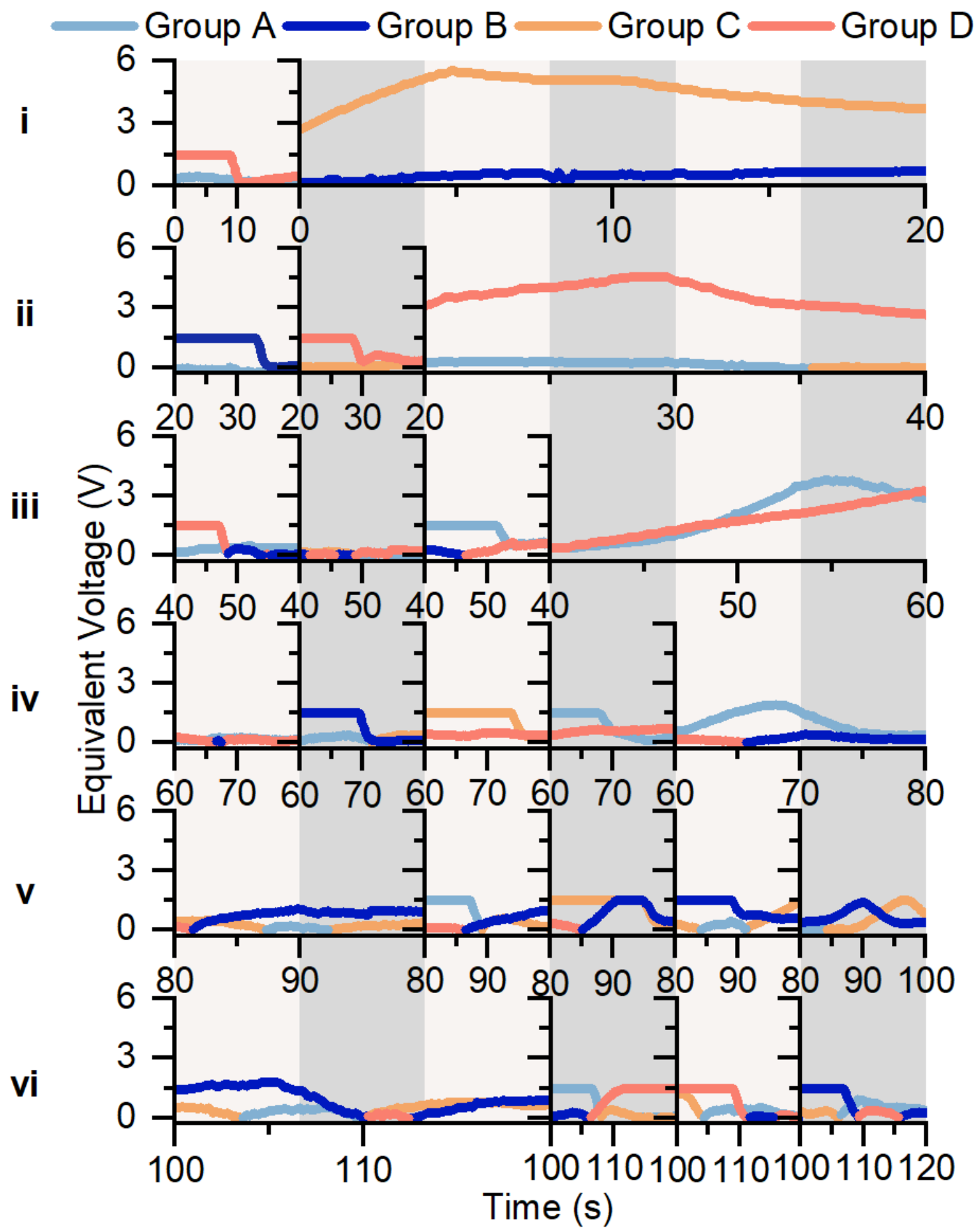

Fig. S11. The actuation of different equivalent segments during the helical propagation of the wave

WBJ-SMA arrangement: from front view, Group A – Left; Group B – Inside; Group C – Right; Group D – Outside

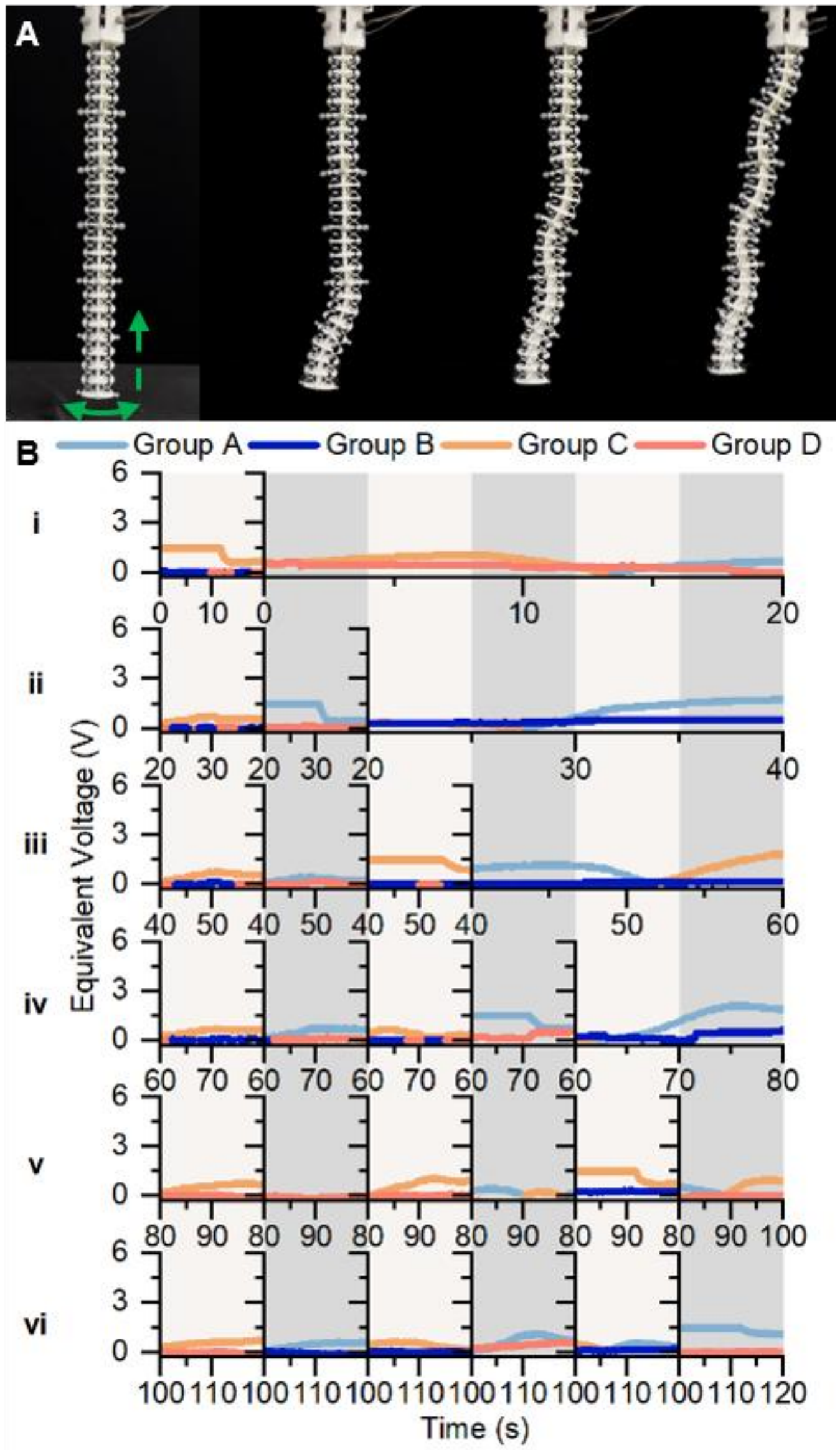

Fig. S12. Sequential motion swinging of the robotic arm

(A) Performance of different equivalent segments during the sequential swinging of the robotic arm and (B) its actuation. WBJ-SMA arrangement: from front view, Group A – Left; Group B – Inside; Group C – Right; Group D – Outside

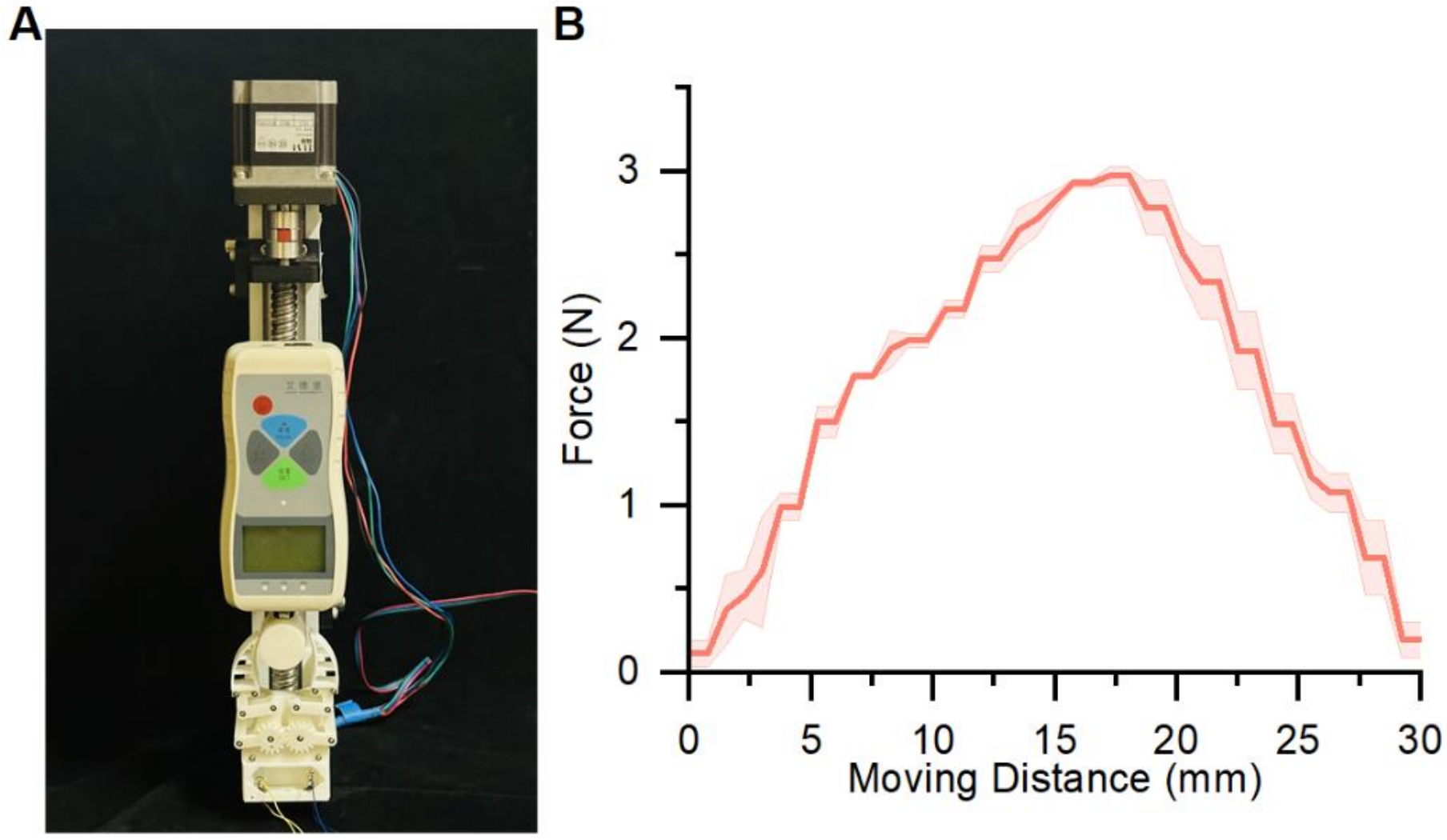

Fig. S13. Force characterization of the soft gripper

(A) Experimental setup. (B) Force response to displacement of the grasped object.

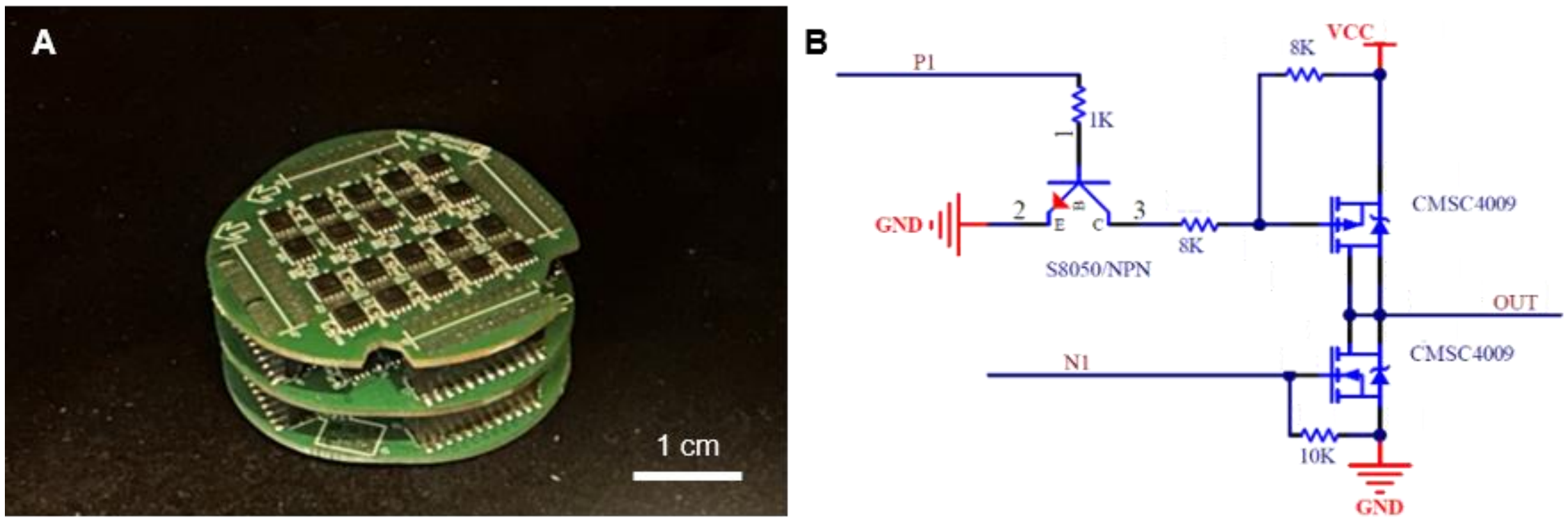

Fig. S14. Miniaturized design of the actuation electronics control

(A) Picture of the actuation board. (B) Basic working actuation unit of the actuation board.

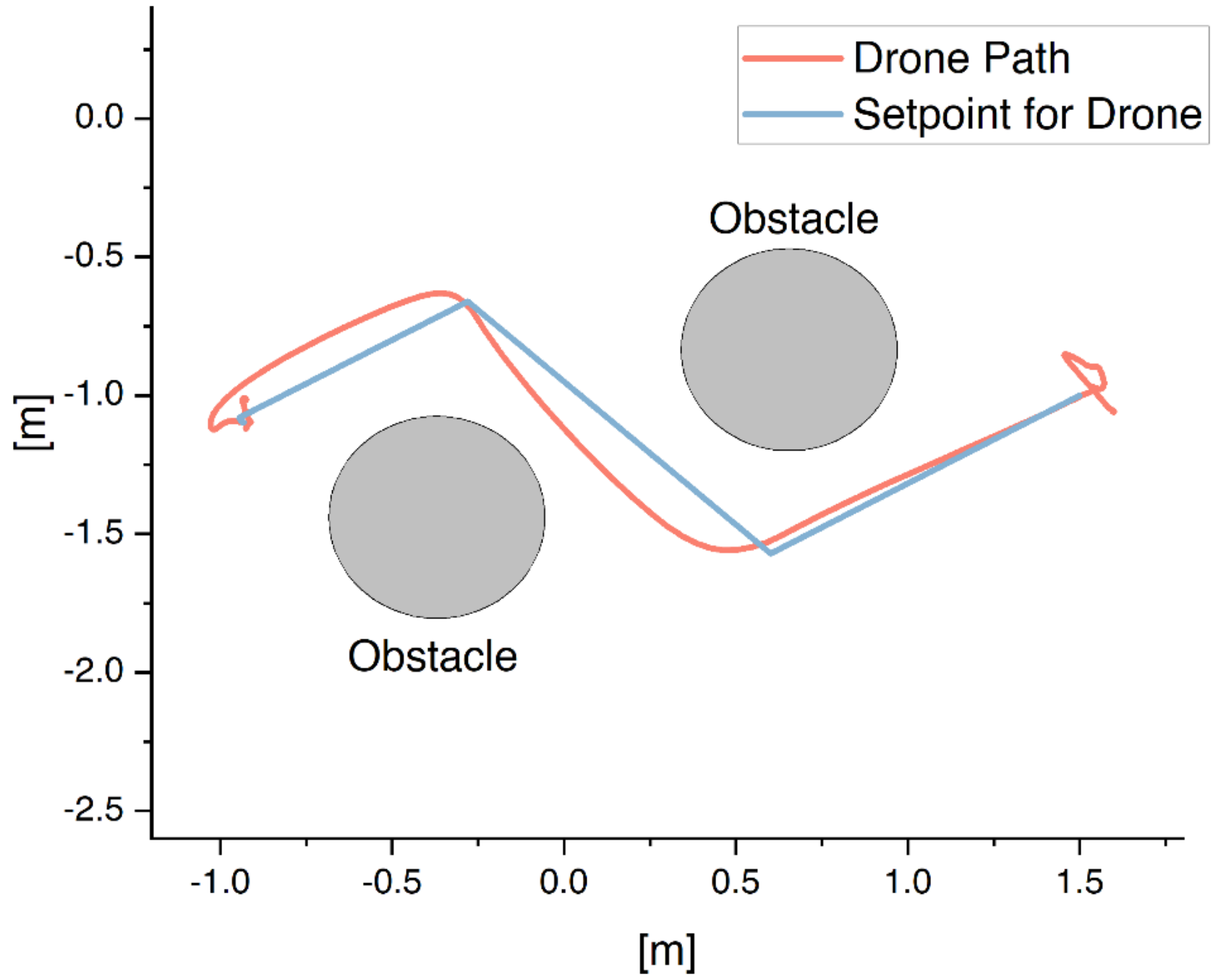

Fig. S15. Planned and the experimental data of the drone route

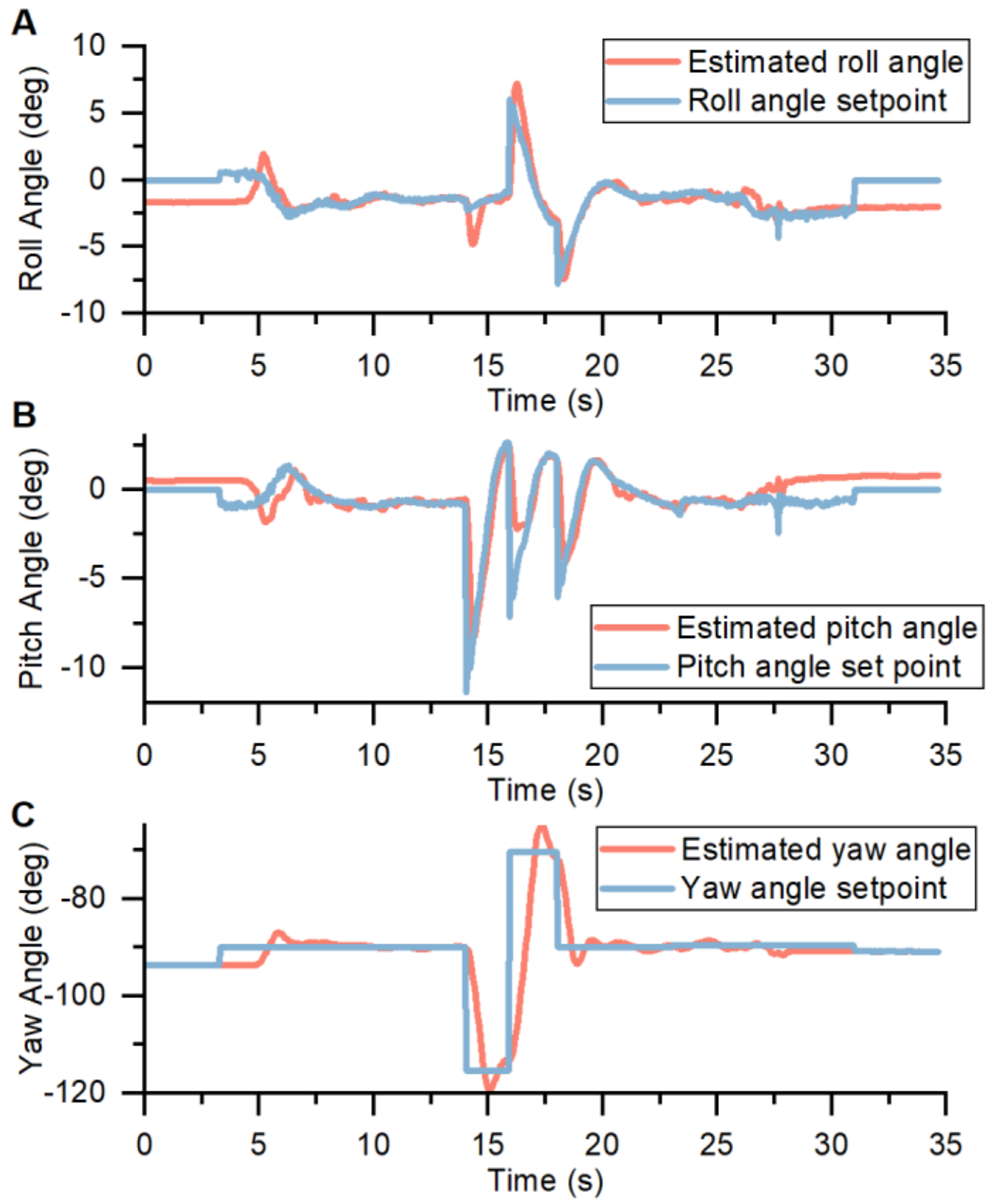

Fig. S16. Set point and experimental data of the drone altitude
(A) Roll angle. (B) Pitch angle. (C) Yaw Angle.

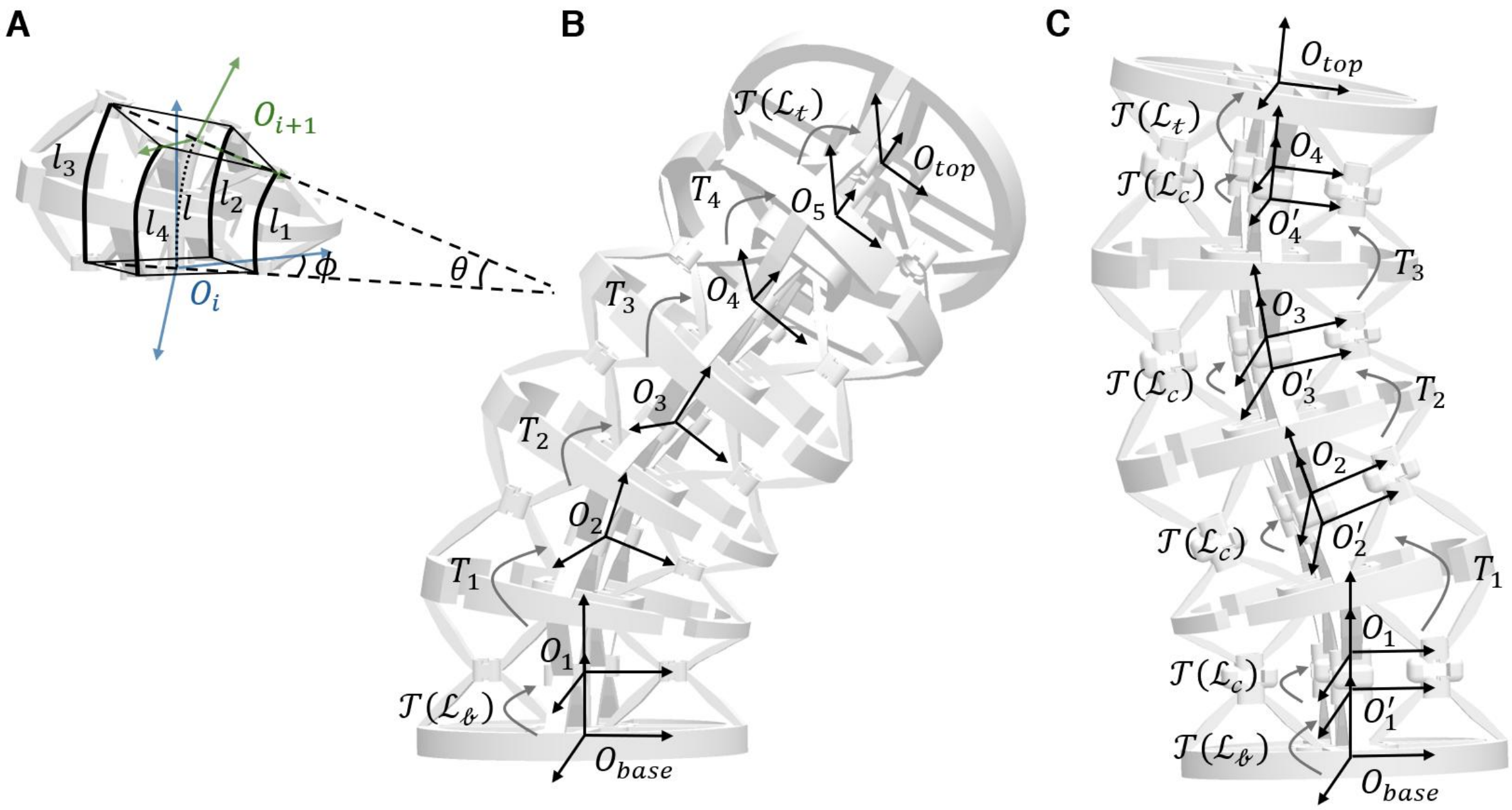

Fig. S17. Schematic of the definition for modelling

(A) Schematic of a single module. (B) Coordinate distribution in the compact arrangement. (C) Coordinate distribution in the stack arrangement.

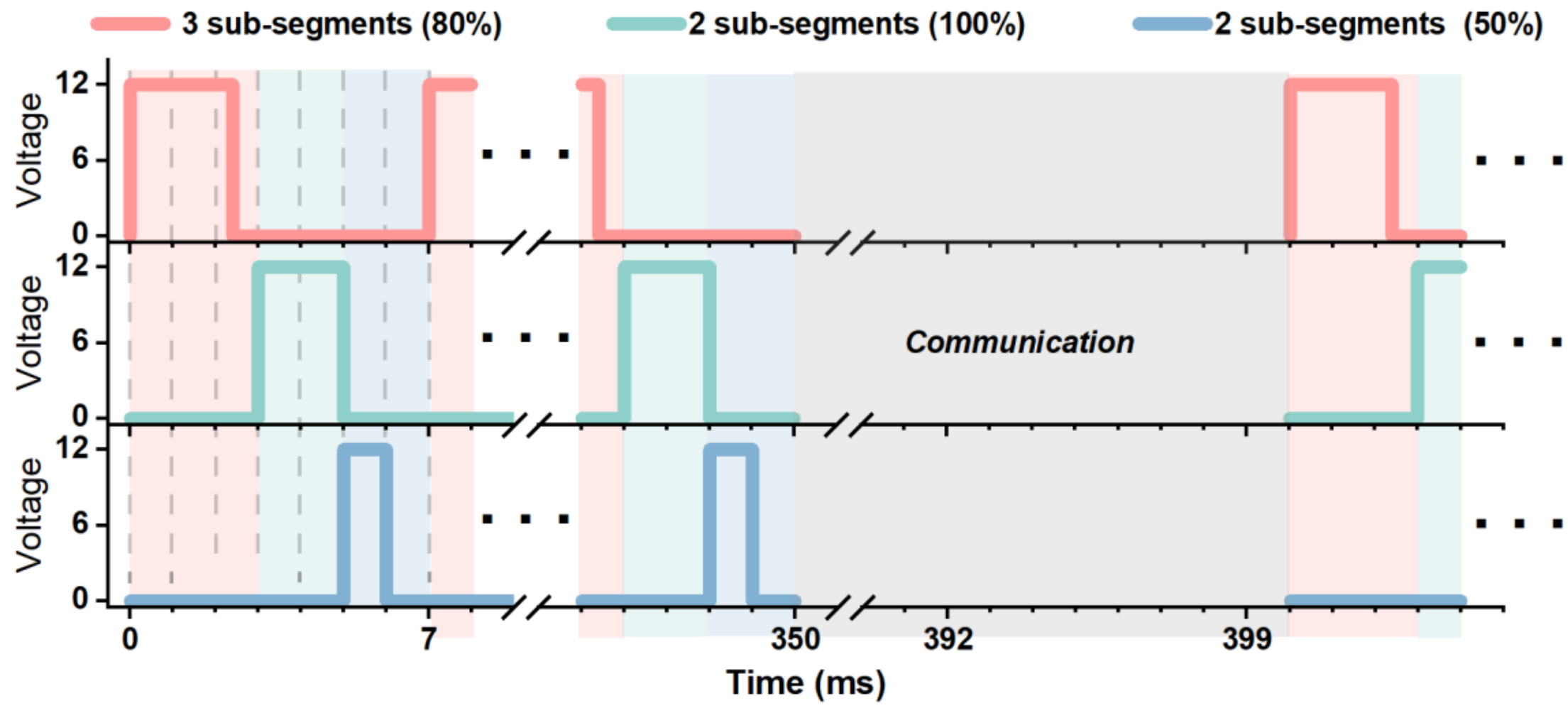

Fig. S18. Actuation protocol of the 7 sub-segment arm

Supplementary Videos

Movie S1. Fabrication of the Wire Bonding Junction addition and removement.

Movie S2. Thermal response of the Wire Bonding Junction addition and removement.

Movie S3. Thermal response differences in bending behavior of compact and stacked arrangements.

Movie S4. Bending experiment with different controlling segments.

Movie S5. Circular path following with different equivalent segments.

Movie S6. Avoidance of different number of obstacles with different equivalent segments.

Movie S7. High DOF arm shape reconfiguration following the drawn curve.

Movie S8. Actuation map of multiple end effectors.

Movie S9. Rolling deployment for extension.

Movie S10. Integrated multi-end-effectors sampling arm deployed by drone.

Movie S11. Sampling task of the dexterous arm.

Movie S12. Spatial deformation of the soft robot arm enabled by WBJ.

Movie S13. Aerial operation of the WBJ robot arm.

REFERENCES

1. G. Sumbre, G. Fiorito, T. Flash, B. Hochner, Motor control of flexible octopus arms. *Nature* **433**, 595–596 (2005).

2. J. N. Richter, B. Hochner, M. J. Kuba, Pull or push? Octopuses solve a puzzle problem. *PLOS ONE* **11**, e0152048 (2016).

3. J. N. Richter, B. Hochner, M. J. Kuba, Octopus arm movements under constrained conditions: adaptation, modification and plasticity of motor primitives. *J. Exp. Biol.* **218**, 1069–1076 (2015).

4. Y. Yekutieli, R. Sagiv-Zohar, R. Aharonov, Y. Engel, B. Hochner, T. Flash, Dynamic model of the octopus arm. I. Biomechanics of the octopus reaching movement. *J. Neurophysiol.* **94**, 1443–1458 (2005).

5. B. Hochner, L. Zullo, T. Shomrat, G. Levy, N. Nesher, Embodied mechanisms of motor control in the octopus. *Curr. Biol.* **33**, R1119–R1125 (2023).

6. T. Flash, L. Zullo, Biomechanics, motor control and dynamic models of the soft limbs of the octopus and other cephalopods. *J. Exp. Biol.* **226**, jeb245295 (2023).

7. C. Laschi, B. Mazzolai, M. Cianchetti, Soft robotics: Technologies and systems pushing the boundaries of robot abilities. *Sci. Robot.* **1**, eaah3690 (2016).

8. S. Kim, C. Laschi, B. Trimmer, Soft robotics: a bioinspired evolution in robotics. *Trends Biotechnol.* **31**, 287–294 (2013).

9. R. Pfeifer, M. Lungarella, F. Iida, Self-Organization, Embodiment, and Biologically Inspired Robotics. *Science* **318**, 1088–1093 (2007).

10. G. Mengaldo, F. Renda, S. L. Brunton, M. Bächer, M. Calisti, C. Duriez, G. S. Chirikjian, C. Laschi, A concise guide to modelling the physics of embodied intelligence in soft robotics. *Nat. Rev. Phys.* **4**, 595–610 (2022).

11. T. Nanayakkara, B. Mulvey, S. Perera, Y. Ge, Z. Yu, P. Sunilkumar, "Soft Robots as a Platform to Understand Embodied Intelligence" in Handbook on Soft Robotics, T. Nanayakkara, Ed. (Springer Nature Switzerland, Cham, 2024; https://doi.org/10.1007/978-3-031-68620-7_3), 35–84.

12. M. Cianchetti, C. Laschi, A. Menciassi, P. Dario, Biomedical applications of soft robotics. *Nat. Rev. Mater.* **3**, 143–153 (2018).

13. W. Xin, F. T. Pan, Y. Li, P. W. Y. Chiu, Z. Li, "A Novel Biomimic Soft Snail Robot Aiming for Gastrointestinal (GI) Tract Inspection" in *2020 8th IEEE RAS/EMBS International Conference for Biomedical Robotics and Biomechatronics (BioRob)* (2020; https://ieeexplore.ieee.org/document/9224300/), pp. 1049–1054.

14. E. Del Dottore, A. Mondini, N. Rowe, B. Mazzolai, A growing soft robot with climbing plant–inspired adaptive behaviors for navigation in unstructured environments. *Sci. Robot.* **9**, eadi5908 (2024).

15. N. R. Sinatra, C. B. Teeple, D. M. Vogt, K. K. Parker, D. F. Gruber, R. J. Wood, Ultragentle manipulation of delicate structures using a soft robotic gripper. *Sci. Robot.* **4**, eaax5425 (2019).

16. J. Shintake, V. Cacucciolo, D. Floreano, H. Shea, Soft Robotic Grippers. *Adv. Mater.* **30**, 1707035 (2018).

17. E. W. Hawkes, L. H. Blumenschein, J. D. Greer, A. M. Okamura, A soft robot that navigates its environment through growth. *Sci. Robot.* **2**, eaan3028 (2017).

18. Z. Chen, D. Wu, Q. Guan, D. Hardman, F. Renda, J. Hughes, T. G. Thuruthel, C. D. Santina, B. Mazzolai, H. Zhao, C. Stefanini, A survey on soft robot adaptability: implementations, applications, and prospects. *IEEE Robot. Autom. Mag.* **33**, 183–195 (2025).

19. A. Mohammad, M. Russo, Y. Fang, X. Dong, D. Axinte, J. Kell, An Efficient Follow-the-Leader Strategy for Continuum Robot Navigation and Coiling. *IEEE Robot. Autom. Lett.* **6**, 7493–7500 (2021).

20. M. Russo, S. M. H. Sadati, X. Dong, A. Mohammad, I. D. Walker, C. Bergeles, K. Xu, D. A. Axinte, Continuum Robots: An Overview. *Adv. Intell. Syst.* **5**, 2200367 (2023).

21. L. Tang, J. Wang, Y. Zheng, G. Gu, L. Zhu, X. Zhu, Design of a cable-driven hyper-redundant robot with experimental validation. *Int. J. Adv. Robotic Syst.* **14**, 172988141773445 (2017).

22. Z. Xie, F. Yuan, J. Liu, L. Tian, B. Chen, Z. Fu, S. Mao, T. Jin, Y. Wang, X. He, G. Wang, Y. Mo, X. Ding, Y. Zhang, C. Laschi, L. Wen, Octopus-inspired sensorized soft arm for environmental interaction. *Sci. Robot.* **8**, eadh7852 (2023).

23. J. Liu, Z. Zhu, L. Wen, Underwater soft arm grasping with simplified control using octopus-inspired bending propagation. *npj Robot.* **4**, 2 (2026).

24. L. Wang, N. Simaan, "Investigation of Error Propagation in Multi-backbone Continuum Robots" in *Advances in Robot Kinematics*, J. Lenarčič, O. Khatib, Eds. (Springer International Publishing, Cham, 2014; https://doi.org/10.1007/978-3-319-06698-1_40), 385–394.

25. C. D. Santina, R. K. Katzschmann, A. Bicchi, D. Rus, Model-based dynamic feedback control of a planar soft robot: trajectory tracking and interaction with the environment. *Int. J. Robot. Res.* **39**, 490–513 (2020).

26. A. D. Marchese, R. K. Katzschmann, D. Rus, A Recipe for Soft Fluidic Elastomer Robots. *Soft Rob.* **2**, 7–25 (2015).

27. L. Shui, L. Zhu, Z. Yang, Y. Liu, X. Chen, Energy efficiency of mobile soft robots. *Soft Matter* **13**, 8223–8233 (2017).

28. M. Cianchetti, A. Licofonte, M. Follador, F. Rogai, C. Laschi, Bioinspired Soft Actuation System Using Shape Memory Alloys. *Actuators* **3**, 226–244 (2014).

29. R. Diogo, V. Abdala, *Muscles of Vertebrates: Comparative Anatomy, Evolution, Homologies and Development* (CRC Press : Science Publishers, Boca Raton, Florida, 2010).

30. K. Stokes, Skeletal muscle: from molecules to movement. *Br. J. Sports Med.* **40**, 950 (2006).

31. G. Karpati, D. Hilton-Jones, K. Bushby, R. C. Griggs, Eds., *Disorders of Voluntary Muscle* (Cambridge University Press, Cambridge, Online-Ausg., 2010).

32. W. M. Kier, M. P. Stella, The arrangement and function of octopus arm musculature and connective tissue. *J. Morphol.* **268**, 831–843 (2007).

33. L. Zullo, S. M. Fossati, P. Imperadore, M.-T. Nödl, Molecular Determinants of Cephalopod Muscles and Their Implication in Muscle Regeneration. *Front. Cell Dev. Biol.* **5**, 53 (2017).

34. H. Matzner, Y. Gutfreund, B. Hochner, Neuromuscular System of the Flexible Arm of the Octopus: Physiological Characterization. *J. Neurophysiol.* **83**, 1315–1328 (2000).

35. N. Nesher, F. Maiole, T. Shomrat, B. Hochner, L. Zullo, From synaptic input to muscle contraction: arm muscle cells of *Octopus vulgaris* show unique neuromuscular junction and excitation–contraction coupling properties. *Proc. R. Soc. B* **286**, 20191278 (2019).

36. N. Feinstein, N. Nesher, B. Hochner, FUNCTIONAL MORPHOLOGY OF THE NEUROMUSCULAR SYSTEM OF THE OCTOPUS VULGARIS ARM. *Vie Milieu* , (2011).

37. L. Zullo, H. Eichenstein, F. Maiole, B. Hochner, Motor control pathways in the nervous system of Octopus vulgaris arm. *J. Comp. Physiol. A* **205**, 271–279 (2019).

38. N. El-Atab, R. B. Mishra, F. Al-Modaf, L. Joharji, A. A. Alsharif, H. Alamoudi, M. Diaz, N. Qaiser, M. M. Hussain, Soft Actuators for Soft Robotic Applications: A Review. *Adv. Intell. Syst.* **2**, 2000128 (2020).

39. D. R. Yao, I. Kim, S. Yin, W. Gao, Multimodal Soft Robotic Actuation and Locomotion. *Adv. Mater.* **36**, 2308829 (2024).

40. S. Zhou, Y. Li, Q. Wang, Z. Lyu, Integrated Actuation and Sensing: Toward Intelligent Soft Robots. *Cyborg Bionic Syst.* **5**, 0105 (2024).

41. C. Cheng, J. Cheng, W. Huang, Design and Development of a Novel SMA Actuated Multi-DOF Soft Robot. *IEEE Access* **7**, 75073–75080 (2019).

42. Z. J. Patterson, A. P. Sabelhaus, K. Chin, T. Hellebrekers, C. Majidi, "An Untethered Brittle Star-Inspired Soft Robot for Closed-Loop Underwater Locomotion" in *2020 IEEE/RSJ International Conference on Intelligent Robots and Systems (IROS)* (2020; https://ieeexplore.ieee.org/document/9341008/), 8758–8764.

43. W. Liu, Z. Jing, J. Huang, X. Dun, L. Qiao, H. Leung, W. Chen, An Inchworm-Snake Inspired Flexible Robotic Manipulator With Multisection SMA Actuators for Object Grasping. *IEEE Trans Ind Electron* **70**, 12616–12625 (2023).

44. C. Della Santina, C. Duriez, D. Rus, Model-Based Control of Soft Robots: A Survey of the State of the Art and Open Challenges. *IEEE Control Syst.* **43**, 30–65 (2023).

45. D. A. Haggerty, M. J. Banks, E. Kamenar, A. B. Cao, P. C. Curtis, I. Mezić, E. W. Hawkes, Control of soft robots with inertial dynamics. *Sci. Robot.* **8**, eadd6864 (2023).

46. E. Almanzor, F. Ye, J. Shi, T. G. Thuruthel, H. A. Wurdemann, F. Iida, Static Shape Control of Soft Continuum Robots Using Deep Visual Inverse Kinematic Models. *IEEE Transactions on Robotics* **39**, 2973–2988 (2023).

47. Z. Tang, L. Tian, W. Xin, Q. Wang, D. Rus, C. Laschi, A general soft robotic controller inspired by neuronal structural and plastic synapses that adapts to diverse arms, tasks, and perturbations. *Sci. Adv.* **12**, eaea3712 (2026).

48. F. Xu, Y. Zhang, J. Sun, H. Wang, Adaptive Visual Servoing Shape Control of a Soft Robot Manipulator Using Bézier Curve Features. *IEEE/ASME Trans. Mechatron.* **28**, 945–955 (2023).

49. X. An, Y. Cui, H. Sun, Q. Shao, H. Zhao, Active-Cooling-in-the-Loop Controller Design and Implementation for an SMA-Driven Soft Robotic Tentacle. *IEEE Trans. Robot.* **39**, 2325–2341 (2023).

50. H. Yang, Z. Tao, J. Yang, W. Ma, H. Zhang, M. Xu, M. Wu, S. Sun, H. Jin, W. Li, L. Wang, S. Zhang, A lightweight prosthetic hand with 19-DOF dexterity and human-level functions. *Nat. Commun.* **16**, 955 (2025).

51. F. Pan, J. Liu, Z. Zuo, X. He, Z. Shao, J. Chen, H. Wang, Q. Zhang, F. Yuan, B. Chen, T. Jin, L. He, Y. Wang, K. Zhang, X. Ding, T. Li, L. Wen, Miniature deep-sea morphable robot with multimodal locomotion. *Sci. Robot.* **10**, eadp7821 (2025).

52. C. Della Santina, A. Bicchi, D. Rus, On an Improved State Parametrization for Soft Robots With Piecewise Constant Curvature and Its Use in Model Based Control. *IEEE Robot. Autom. Lett.* **5**, 1001–1008 (2020).

53. R. J. Webster, B. A. Jones, Design and kinematic modeling of constant curvature continuum robots: A review. *Int. J. Robot. Res.* **29**, 1661–1683 (2010).
